# Electrochemical CO_2_ Reduction on a Bi–Sn Eutectic Alloy in Acidic Media for Formic Acid Production

**DOI:** 10.1002/cssc.202502541

**Published:** 2026-04-21

**Authors:** Avni Guruji, Alejandro Cañete‐Arché, Yuvraj Y. Birdja, Ranjith Prasannachandran, Max García‐Melchor, Deepak Pant

**Affiliations:** ^1^ Electrochemistry Excellence Centre (ELEC) Materials & Chemistry Unit Flemish Institute for Technological Research (VITO) Mol Belgium; ^2^ Dipartimento di Scienza Applicata e Tecnologia Torino Italy; ^3^ School of Chemistry Trinity College Dublin College Green Dublin D02 PN40 Ireland; ^4^ Center for Cooperative Research on Alternative Energy (CIC energiGUNE) Basque Research and Technology Alliance (BRTA) C/ Albert Einstein 48, 01510 Vitoria‐Gasteiz Spain; ^5^ IKERBASQUE Basque Foundation for Science Plaza de Euskadi 5, 48009 Bilbao Spain; ^6^ Center for Advanced Process Technology for Urban Resource Recovery (CAPTURE) Zwijnaarde Belgium

**Keywords:** bimetallic catalysts, DFT calculations, electrochemical CO_2_ reduction, formic acid, gas‐diffusion electrode

## Abstract

Electrochemical CO_2_ reduction (eCO_2_R) offers a sustainable route for carbon utilization, but most electrolyzers operate in neutral or alkaline media, where (bi)carbonate formation limits long‐term operation and complicates product recovery. Here, we show that operating eCO_2_R under acidic conditions enables direct formic acid production while minimizing (bi)carbonate accumulation. A eutectic Bi–Sn gas‐diffusion electrode (GDE) achieved a faradaic efficiency (FE) of 81.3% toward formic acid at −100 mA cm^−2^ and in a pH 3 electrolyte, outperforming Bi and Sn GDEs, with formic acid remaining the dominant product up to −400 mA cm^−2^. Density functional theory calculations revealed a synergistic Bi–Sn interfacial effect, where weakened hydrogen adsorption and intermediate binding of CO_2_‐to‐formate intermediates collectively suppress hydrogen evolution and promote formic acid formation. The GDE maintained stable performance with <10% FE loss in a 100 h continuous operation, using a periodic electrolyte replacement strategy. These results establish acidic eCO_2_R as a viable strategy for high‐purity formic acid production and demonstrate how interfacial alloy engineering can advance CO_2_ electrolysis toward scalable, renewable energy‐powered chemical manufacturing.

## Graphical abstract

Acidic CO_2_ electrolysis enables direct formic acid production with minimal (bi)carbonate formation. A eutectic Bi–Sn gas‐diffusion electrode achieves 81.3% faradaic efficiency (FE) at −100 mA cm^−2^ and pH 3, retaining selectivity up to −400 mA cm^−2^ and stable operation for 100 h with < 10% FE loss. Density functional theory reveals synergistic Bi–Sn interfaces that weaken hydrogen adsorption while stabilizing formate, thereby suppressing hydrogen evolution reaction.

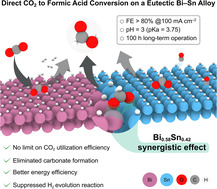

## 1 Introduction

Over the past decades, carbon dioxide reduction has been a central focus of both academic and industrial research as a strategy to mitigate greenhouse gas emissions and reduce reliance on fossil carbon feedstocks. Various approaches, including photochemical, thermal, biological, plasma‐assisted, and electrochemical methods, have been explored to convert thermodynamically stable CO_2_ into value‐added fuels and chemicals [1, 2]. Among these, electrochemical CO_2_ reduction (eCO_2_R) stands out for its operation under mild conditions and compatibility with renewable electricity, enabling the storage of intermittent solar or wind power in chemical form. eCO_2_R proceeds through complex electron–proton transfer reactions that yield a range of carbon‐based products, including single‐carbon (C_1_) molecules such as formic acid (HCOOH) and methanol (CH_3_OH), and multicarbon (C_2+_) products such as ethylene (C_2_H_4_) and propanol (C_3_H_8_O) [3].

Within this spectrum, CO and HCOOH are particularly attractive because their formation involves only two‐electron transfer steps, simplifying reaction pathways and enabling higher selectivity [4, 5]. Formic acid has emerged as one of the most commercially viable CO_2_‐derived products, supported by an established market in the leather, chemical, pharmaceutical, and agricultural sectors [6, 7]. Its liquid handling, potential as a hydrogen carrier, and suitability for direct formic acid fuel cells further enhance its industrial appeal [8, 9]. Techno‐economic assessments identify formic acid as an early commercialization candidate owing to its favorable energy efficiency and near‐commercial readiness (TRL 7–8) [5].

Despite these advances, even the most efficient eCO_2_R systems for HCOOH production, with faradaic efficiencies (FEs) exceeding 90% [10, 11, 12, 13], remain far from practical deployment because strong local alkalinity is typically required. Instead of being reduced [14, 15], a significant fraction of the CO_2_ dissolved in the electrolyte reacts with hydroxide ions to form carbonate species, as shown in Equation (1):

(1) $$2{\mathrm{OH}}_{\left(\mathrm{aq}\right)}^{-}+{\mathrm{CO}}_{2\left(g\right)}\to {\mathrm{CO}}_{3\left(\mathrm{aq}\right)}^{2-}+H_{2}O_{\left(l\right)}$$

This side reaction limits the theoretical carbon utilization efficiency to 50% for two‐electron processes, making CO_2_‐to‐HCOOH conversion inefficient. Moreover, regenerating CO_2_ from carbonate is highly energy‐intensive [15, 16, 17]. A further complication arises because the main product in alkaline conditions is formate (HCOO^–^) rather than formic acid, which predominates only below its pKa of 3.75 [18]. Converting formate to formic acid therefore requires strong acids, producing inorganic salt by‐products and increasing downstream processing costs.

**FIGURE 1:**
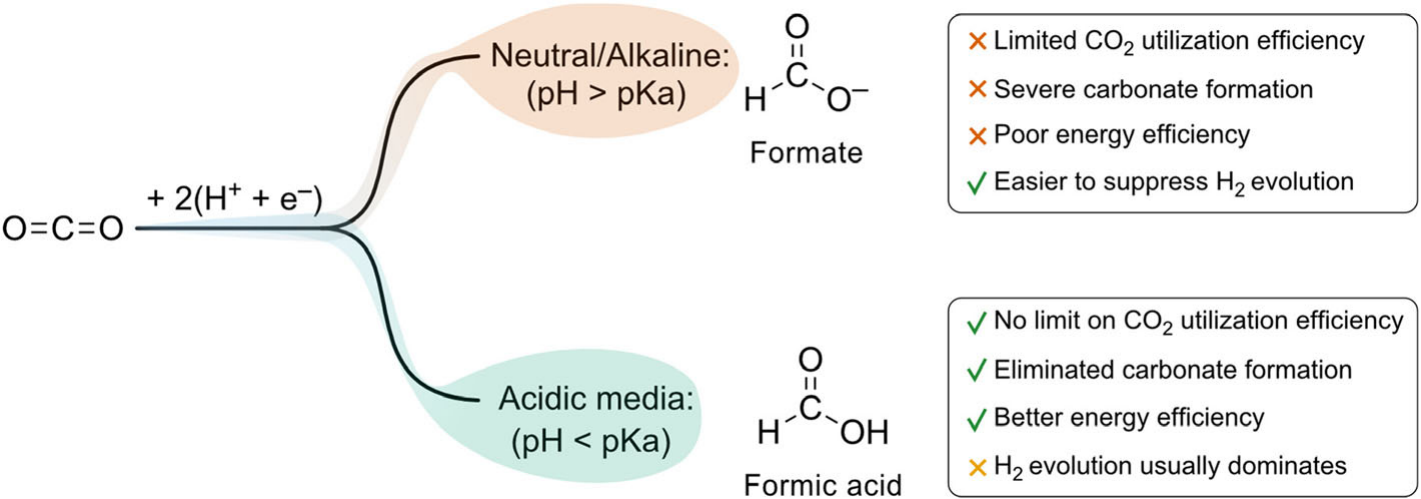
Schematic illustration of electrochemical CO_2_ reduction to formate or formic acid as a function of electrolyte pH, highlighting the main advantages and challenges in each regime.

In contrast, eCO_2_R in acidic media can overcome these limitations (Figure 1). When hydronium ions (H_3_O^+^) act as the proton source, no hydroxide ions are generated, allowing CO_2_ reduction without carbonate formation [19]. Direct formic acid formation also simplifies purification by standard downstream techniques such as distillation, extraction, or electrodialysis. However, the high H_3_O^+^ concentration in acidic electrolytes strongly promotes the hydrogen evolution reaction (HER), often compromising eCO_2_R selectivity [16, 20, 21]. Achieving high eCO_2_R activity in acidic environments while suppressing HER therefore remains a major challenge.

Recent strategies have focused on catalyst and electrolyte design to mitigate HER and promote selective eCO_2_R. Approaches such as introducing alkali cations or tailoring catalyst surfaces can create locally alkaline microenvironments that favor eCO_2_R over HER. Among available catalysts, bismuth (Bi) and tin (Sn) stand out due to their earth abundance, low toxicity and cost, and intrinsic selectivity toward formic acid [21, 22, 23]. For example, Sun et al. reported Sn single‐atom catalysts with 1 M K^+^ (K_2_SO_4_) electrolyte for efficient CO_2_‐to‐HCOOH conversion at pH 3, achieving a FE of 90.8% at −100 mA cm^−2^ [24], while Qiao and co‐workers demonstrated Bi nanosheets in acidic media reaching 92.2% FE at −237 mA cm^−2^ [25]. However, Bi–Sn binary catalysts, known for exceptional activity and selectivity in alkaline eCO_2_R, remain largely unexplored under acidic conditions.

In alkaline environments, Bi–Sn interfaces outperform many bimetallic systems in formate activity and selectivity, presumably because they weaken hydrogen adsorption [24, 26]. The interfacial synergy between Bi and Sn enhances CO_2_ conversion beyond that of either pure metal or alloy alone. In this context,the eutectic Bi–Sn composition (Bi_0.58_Sn_0.42_, where subscripts denote weight fractions) is particularly attractive because its phase‐separated microstructure exhibits a small characteristic grain size and a high density of Bi–Sn boundaries [26, 27, 28]. Unlike off‐eutectic compositions, the eutectic solidifies into a fully lamellar structure of alternating Bi and Sn phases, providing a comparatively high interfacial area. This structure not only creates abundant catalytic boundaries but also enables low‐temperature fabrication, as the Bi–Sn eutectic melts at ≈139°C, far below the melting points of pure Bi (271°C) and Sn (232°C), facilitating energy‐efficient and scalable catalyst fabrication [29]. Tang et al. showed that this composition offers superior formate selectivity compared with off‐eutectic alloys, while Pb–Bi–Sn eutectic systems demonstrate enhanced formate production and suppressed HER a [26, 30]. However, whether this eutectic‐driven performance extends to acidic media, and whether interfacial synergy persists under these conditions, remain unexplored.

In this work, we report for the first time a Bi–Sn eutectic‐based gas‐diffusion electrode (GDE) for eCO_2_R in acidic media, enabling direct conversion to formic acid. Systematic evaluation across current densities from −100 mA cm^−2^ to −400 mA cm^−2^ and electrolyte pH 1–3 revealed that while HER dominates at pH 1 and remains significant at pH 2, formic acid becomes the major product at pH 3 even at industrially relevant current densities (≤300 mA cm^−2^). Under these conditions, the Bi_0.58_Sn_0.42_ GDE achieved a maximum FE of 81.26 ± 3.06% at −100 mA cm^−2^, outperforming Bi (68.63 ± 2.53%) and Sn (77.02 ± 4.47%) benchmarks, and maintained performance over 100 h with less than 20% FE loss. Density functional theory (DFT) calculations uncovered a synergistic Bi–Sn interfacial effect, in which the alloy weakens hydrogen adsorption relative to either metal while maintaining intermediate binding strengths for formate and carboxyl intermediates. This balance suppresses HER and CO formation, steering selectivity toward formic acid. Together, these results identify the Bi_0.58_Sn_0.42_ GDE as a promising candidate for addressing the long‐standing challenges in acidic eCO_2_R and provide the first mechanistic insight into how eutectic composition enhances catalytic performance under these conditions.

## 2 Results and Discussion

### 2.1 Electrochemical Reduction of CO_2_

**FIGURE 2:**
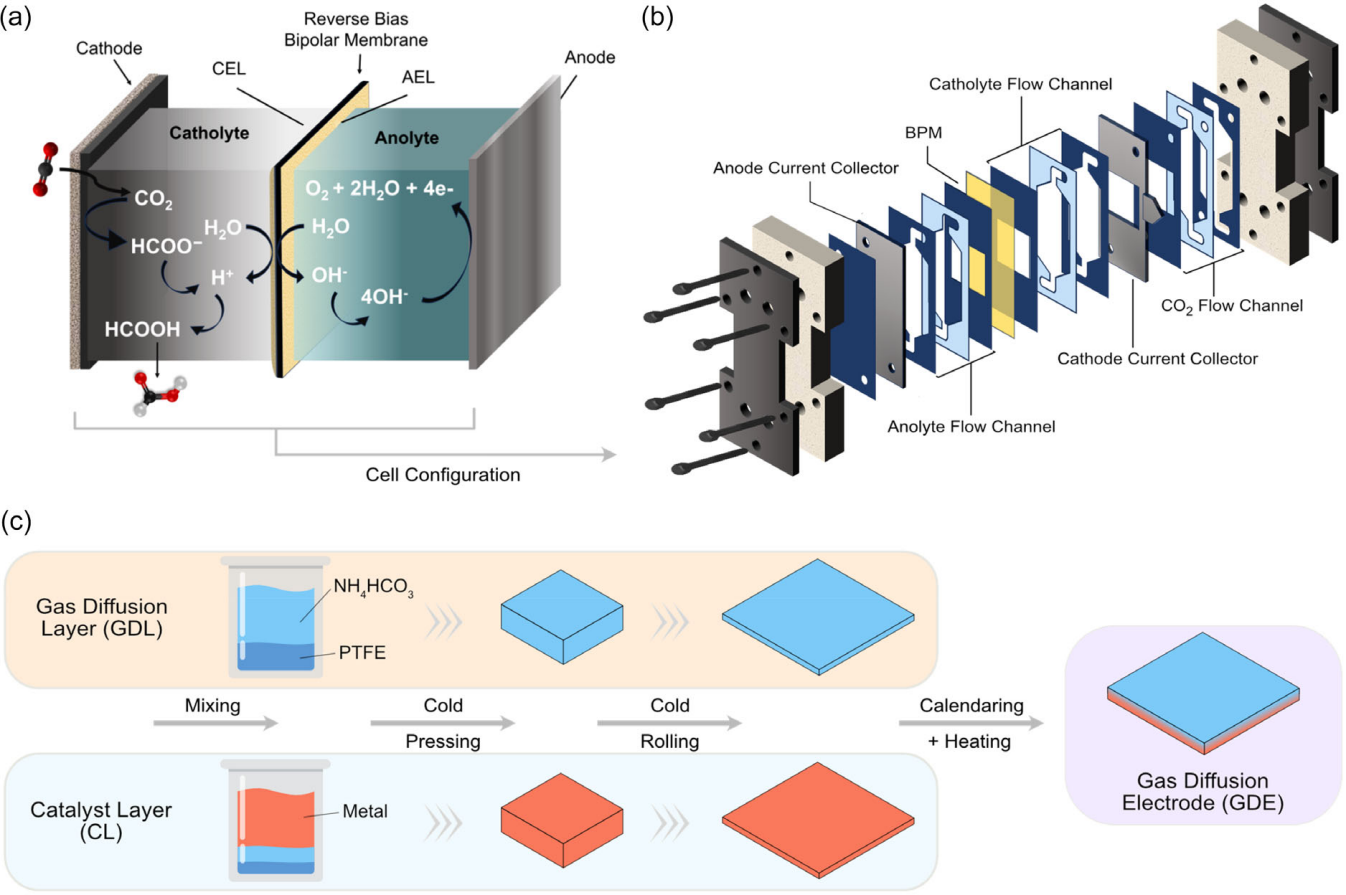
Overview of the acidic eCO_2_R experimental setup. (a) Schematic of eCO_2_R to formic acid and ion transport in a reverse‐biased bipolar‐membrane cell. (b) Exploded view of the micro flow cell configuration used in this work. (c) GDE fabrication process: the gas‐diffusion layer (GDL; NH_4_HCO_3_/polytetrafluoroethylene (PTFE) = 70:30, w/w) and catalyst layer (CL; metal/NH_4_HCO_3_/PTFE = 70:20:10, w/w/w) were prepared separately, compacted by hydraulic pressing, rolled into sheets, laminated by calendaring, and consolidated by 70°C heat treatment.

Acidic eCO_2_R experiments were carried out in a flow‐cell electrolyzer equipped with a bipolar membrane (BPM) operated in reverse bias (Figure 2a). This configuration sustained an acidic environment at the cathode and an alkaline environment at the anode, while enabling ion transport through water dissociation at the BPM interface. The system, illustrated in Figure 2b, allowed continuous CO_2_ feed and efficient electrolyte circulation, providing the mass‐transport conditions required for high‐rate operation. Catalyst electrodes were prepared using the stepwise process summarized in Figure 2c, in which the gas‐diffusion and catalyst layers were fabricated separately and then laminated by calendaring and mild heat treatment. This procedure yielded a carbon‐free, two‐layer GDE consisting of a hydrophobic gas‐diffusion layer and a hydrophilic catalyst layer, sustaining a stable gas–liquid–solid interface for CO_2_ transport to the active surface. Additional operational and manufacturing details are provided in the Experimental Section.

**FIGURE 3:**
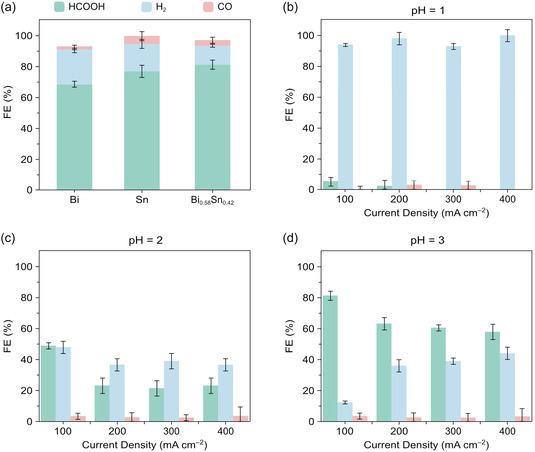
FEs toward H_2_ and eCO_2_R products. (a) Comparison of the catalytic performance of Bi, Sn, and Bi_0.58_Sn_0.42_ GDEs at −100 mA cm^−2^ and pH 3. (b–d) FEs of the Bi_0.58_Sn_0.42_ GDE at current densities of −100 – −400 mA cm^−2^ under pH 1, 2, and 3, respectively. Values represent the mean of three independent measurements; error bars indicate the corresponding standard deviation.

The electrocatalytic performance of Bi, Sn, and eutectic Bi_0.58_Sn_0.42_ GDEs toward eCO_2_R was evaluated under identical galvanostatic conditions (−100 mA cm^*−*2^, pH 3; Figure 3a). Among the three catalysts, Bi_0.58_Sn_0.42_ exhibited the highest selectivity, achieving a formic acid FE of 81.26 ± 3.06%, surpassing Bi (68.63 ± 2.53%) and Sn (77.02 ± 4.47%). Based on these results, subsequent experiments focused on Bi_0.58_Sn_0.42_ across acidic catholytes of pH 1, 2, and 3 under galvanostatic operation at current densities of −100, −200, −300, and −400 mA cm^*−*2^.

At pH 1 (Figure 3b), HER dominated across the entire current‐density range, with formic acid FE below 10%. At pH 2 (Figure 3c), comparable FEs for formic acid and H_2_ were observed at −100 mA cm^*−*2^, while HER prevailed at higher currents. In contrast, at pH 3 (Figure 3d), formic acid became the dominant product at all current densities, reaching its maximum FE at −100 mA cm^*−*2^. These observations underscore the strong dependence of formic acid selectivity on pH: strongly acidic media promote HER, whereas mildly acidic conditions (pH 3), below the pKa of HCOOH (3.75), enable selective eCO_2_R to formic acid, particularly at moderate current densities. The corresponding cell and working‐electrode potential profiles for all investigated pH and current‐density combinations are provided in Figure S5.

### 2.2 Origin of Enhanced Formic Acid Selectivity on the Eutectic Bi–Sn Alloy

To rationalize the enhanced catalytic performance of the Bi_0.58_Sn_0.42_ GDE, we performed periodic DFT calculations to evaluate the thermodynamics of the competing reaction pathways under the experimental conditions. To model these pathways, we characterized the Bi, Sn and Bi–Sn catalysts to identify the most relevant facets and surface structures for a representative model. The planes observed by X‐ray diffraction (XRD; Figure S4) before and after 1 h of electrolysis agreed closely with surface‐stability trends predicted by DFT (Table S3). Detailed peak assignments and phase identification are provided in Figure S4. For Sn, the lowest‐energy surfaces (200) and (101) corresponded to the most intense XRD reflections, followed by (301), (211), and (220). For Bi, both XRD and DFT pointed to (012) and (110) as the most dominant facets, while higher‐energy surfaces such as (104) and (202) were less exposed. This agreement indicates that the electrode surfaces grew under thermodynamic control, supported by the equilibrium Wulff construction shown in Figure S7. Accordingly, the eCO_2_R mechanisms for Bi and Sn were investigated on Bi(012) and Sn(200) surface slabs.

**FIGURE 4:**
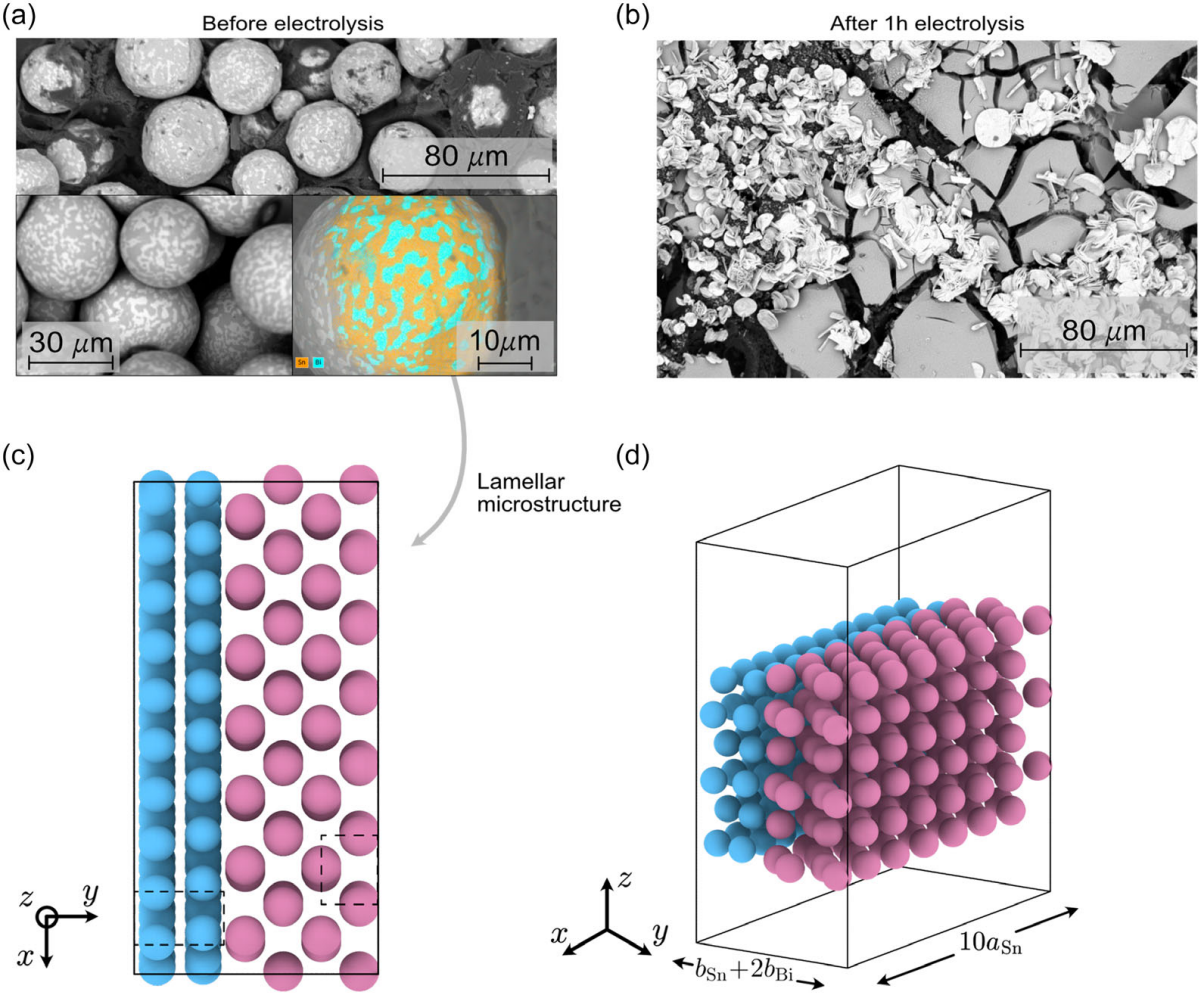
SEM images of the Bi_0.58_Sn_0.42_ GDE illustrating surface morphology before (a) and after 1 h (b) eCO_2_R. Panel (a) includes a higher magnification inset to visualize the lamellar microstructure and a second inset (bottom right) showing an EDX dot map of Bi and Sn overlaid on a representative particle (scale bars as indicated). Structural model of the Bi–Sn heterostructure viewed from (c) the top and (d) a side‐diagonal perspective. Dashed lines in panel (c) denote the unit cells of Sn(200) and Bi(012). Panel (d) follows the same orientation convention as Figure S9. Color code: Bi (pink), Sn (blue).

Structural characterization of the Bi_0.58_Sn_0.42_ GDE confirms a phase‐separated eutectic composition. XRD patterns (Figure S4) display distinct reflections corresponding to rhombohedral Bi and tetragonal β‐Sn, in agreement with the Bi–Sn phase diagram [31, 32]. Scanning electron microscopy (SEM), together with energy‐dispersive X‐ray spectroscopy (EDX), of the as‐prepared electrode (Figure 4a) reveals a lamellar microstructure characteristic of eutectic solidification, consistent with previous reports on this system [26]. Notably, Bi‐ and Sn‐rich lamellae form extended micrometer‐scale domains, with phase boundaries clearly resolved in SEM–EDX elemental maps acquired at 30 µm and, more prominently, 10 µm length scales (bottom‐right inset). The coexistence of Bi‐ and β‐Sn‐rich domains indicates that the catalyst remains phase‐separated rather than forming an intermetallic compound. Based on these observations, the eutectic surface was modeled as an in‐plane Bi–Sn heterostructure rather than a random substitutional alloy. The interface was constructed by joining *p*(10 × 1) − Sn(200) and *p*(7 × 2) − Bi(012) commensurate supercells matched along the y‐direction (Figure 4c,d; see Supporting Information for details).

While the pre‐electrolysis GDE consists of distinct spherical eutectic particles, SEM imaging after 1 h of electrolysis (Figure 4b) showed that these initially well‐defined Bi–Sn boundaries become obscured. This indicates an evolution of the electrode morphology under reaction conditions that hinders the exposure of spherical particles. Thus, the mechanistic interpretation presented herein refers to the initial, precatalyst state that governs early eCO_2_R performance [33]. SEM images of the Bi_0.58_Sn_0.42_ GDE acquired at varying magnifications, illustrating the electrode surface before and after 1 h electrolysis are provided in Figure S2. SEM images of the individual Bi and Sn GDEs can be found in Figure S3.

**FIGURE 5:**
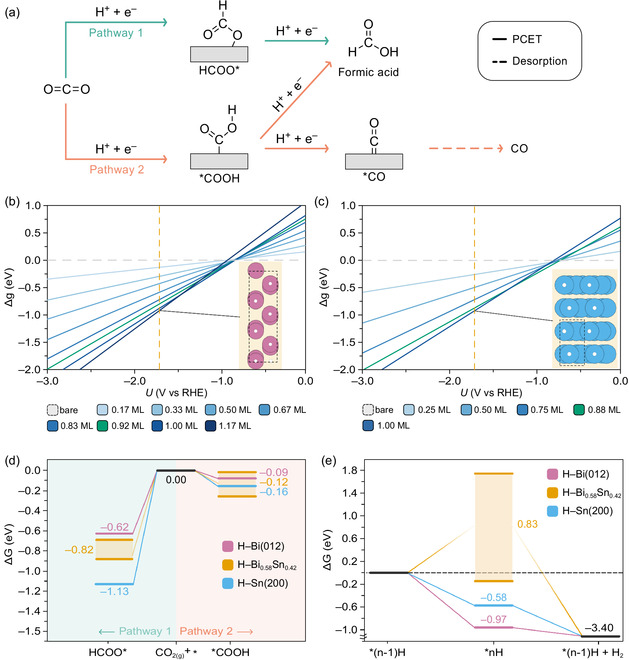
Competing pathways to formic acid and the role of hydrogen coverage on Bi‐ and Sn‐based GDEs. (a) Schematic of the investigated eCO_2_R mechanisms: Pathway 1 proceeds via HCOO* and, after a PCET, yields formic acid, which then desorbs. Pathway 2 proceeds via *COOH, where a PCET step can either generate formic acid or produce *CO, which can desorb or be further reduced on the electrode surface. (b,c) Surface coverage analysis for (b) Bi(012) and (c) Sn(200). The relative Gibbs adsorption energy per surface atom, Δg(U), for *H is plotted against applied potential vs RHE. Surface coverages are labeled according to the IUPAC definition of a monolayer [34], darker shades indicate higher coverages, and the gray dashed line corresponds to the bare surface. The vertical orange dashed line marks –1.70 V_RHE_ (–2.07 V vs Ag/AgCl), which gives the highest FE at –100 mA cm^−2^ and pH 3. Insets show the ground‐state *H configurations at this potential. Bi was modeled as a *p*(3 × 1) − (012) surface slab, and Sn(200) with supercells chosen to finely sample coverages. Green lines indicate the *H coverages used for subsequent adsorption‐energy calculations of HCOO* and *COOH. (d) Gibbs energy diagrams for the eCO_2_R pathways on the hydrogen‐covered Bi(012), Sn(200), and Bi_0.58_Sn_0.42_ surfaces predicted in panels (b) and (c) at –1.70 V_RHE_. (e) Gibbs energies for *H adsorption at –1.70 V_RHE_ on Bi(012), Sn(200), and Bi_0.58_Sn_0.42_. Values represent the energy change upon adding one hydrogen atom to a surface already containing n – 1 adsorbed H atoms.

For the DFT calculations, two eCO_2_R mechanisms were considered (Figure 5a), differing in whether the C or O atom in CO_2_ is hydrogenated first. In Pathway 1, the first proton‐coupled electron transfer (PCET) at the C atom results in the formation of the formate radical anion HCOO* (where * denotes a surface site), while in Pathway 2, the O atom is hydrogenated first, forming the carboxyl radical intermediate *COOH. Because the reaction occurs in acidic media and at cathodic potentials, we explicitly considered *H surface coverages under these conditions [35]. Using systematic enumeration of symmetrically distinct adsorption sites and different *H densities, we identified the resting state for each cathode by plotting relative Gibbs energies as a function of potential. The resulting surface phase diagrams (Figure 5b,c) show that *H coverages on both Bi(012) and Sn(200) surfaces become increasingly favorable at more negative potentials. Under experimental conditions corresponding to the highest FE (–1.7 V vs the reversible hydrogen electrode, V_RHE_), the predicted ground‐state coverages were 1.17 monolayers (ML) for Bi(012) and 1.00 ML for Sn(200), with the respective structures shown in the insets of Figure 5b,c. These configurations consist of six *H atoms occupying all *ontop* sites plus one *bridge* site of the *p*(3 × 1) − Bi(012) slab, and full occupation of *ontop* sites in the *p*(1 × 1) − Sn(200) surface (see Table S5 for details).

After identifying the resting states, we evaluated the most stable binding sites for HCOO* and *COOH. Both intermediates preferentially adsorb on the same *ontop* sites as *H (see Experimental Section for sampling details). To model their adsorption, we removed one *H atom from an *ontop* site. This choice is justified because at –1.70 V_RHE_ slightly lower *H coverages lie within $k_{B}T$ (at 298 K) of the ground‐state coverages (1.17 ML on Bi and 1 ML on Sn) and can therefore coexist according to a Boltzmann distribution, and because HER turnover is expected to intermittently create vacant *ontop* sites. The representative coverages used for modeling eCO_2_R reactivity were 0.92 ML for Bi(012) and 0.88 ML for Sn(200), corresponding to one *ontop* vacancy in the *p*(3 × 2) and *p*(2 × 2) supercells, respectively (green lines in Figure 5b,c).

With the relevant surfaces and hydrogen coverages established, we computed the Gibbs energy profiles for the competing pathways (Figure 5d). DFT calculations revealed that Pathway 1 (via HCOO*) is the most thermodynamically favorable route on both Bi(012) and Sn(200), in agreement with the experimental observation of formic acid as the major product (Figure 3a). The relative stability of HCOO* also serves as a selectivity descriptor. On Sn(200), HCOO* is stabilized by –1.13 eV compared with –0.62 eV on Bi(012). This deeper stabilization on Sn(200) makes the backward reaction to * + CO_2_ highly unfavorable, explaining the higher FE for formic acid on Sn. In contrast, Pathway 2 (via *COOH) is nearly thermoneutral on Bi(012) but more exergonic on Sn(200), consistent with the higher CO selectivity of Sn GDEs.

For the Bi_0.58_Sn_0.42_ heterostructure, the Gibbs energies of both HCOO* and *COOH fell between those of pure Bi(012) and Sn(200) (Figure 5d). To reflect the distribution of adsorption energies arising from different local environments on the Bi_0.58_Sn_0.42_ surface, we report the upper and lower bounds along with the mean values. While HCOO* binding is weakened compared with Sn(200), it remains stronger than on Bi(012), making the backward reaction to * + CO_2_ highly unfavorable and supporting the high FE for formic acid. Similarly, *COOH binding decreases compared with Sn(200) but remains stronger than on Bi(012), explaining the intermediate FE for CO relative to the pure phases, with the decrease in CO production contributing to the enhanced formic acid yield.

The competition between formic acid production and HER can be rationalized by examining adsorption of *H and HCOO* on the catalyst resting states (insets of Figure 5b,c). Starting from the hydrogenated surfaces with one vacancy, we evaluated whether a PCET favors formation of a fully (saturated) H‐covered surface or HCOO* adsorption. The adsorption energies for *H at the experimental potential corresponding to the highest FE (–1.7 V_RHE_; Figure 5e) are –0.97 eV (Bi, *ontop*), –0.58 eV (Sn, *ontop*), and + 0.83 eV (Bi_0.58_Sn_0.42_, *ontop*), while those for HCOO* are –0.62 eV (Bi, *ontop*), –1.13 eV (Sn, *ontop*), and –0.82 eV (Bi_0.58_Sn_0.42_, *ontop*) (Figure 5d).

These results reveal a clear trend. On Bi(012), *H adsorption dominates over HCOO*, consistent with higher HER activity. On Sn(200), HCOO* is favored, in line with higher selectivity for formic acid compared with Bi(012). On Bi_0.58_Sn_0.42_, the difference is further increased: *H adsorption becomes thermodynamically unfavorable (+0.83 eV), while HCOO* remains strongly stabilized (–0.82 eV). Across the different local domains of the alloy, the Gibbs energies of HCOO* adsorption vary modestly among Bi, Sn, and Bi–Sn sites (ranging –0.69 to –0.88 eV), whereas *H adsorption differs substantially. In particular, *H binding is strongly destabilized at interfacial Bi sites, showing the largest gap between *H and *HCOO adsorption (2.58 eV), followed by Sn and Bi–Sn sites (0.92 eV and 0.71 eV, respectively).

This energetic shift explains why the Bi_0.58_Sn_0.42_ GDE suppresses HER relative to Bi and Sn GDEs while promoting formic acid formation. In acidic media, HER can follow a Volmer–Heyrovský sequence (*H + e^–^ → *H; *H + H^+^ + e^–^ → H_2_ + *). On Bi(012) and Sn(200), Volmer is exergonic (Figure 5e), driving *H coverage toward saturation and thereby favoring the subsequent Heyrovský reaction. In contrast, the Volmer step on Bi_0.58_Sn_0.42_ is, on average, endergonic under the optimal experimental conditions, making a saturated *H coverage unlikely and thus largely suppressing the Heyrovský route. At the same time, HCOO* remains favorably stabilized on Bi_0.58_Sn_0.42_, biasing the available sites toward eCO_2_R rather than further hydrogenation.

### 2.3 Durability of the Bi–Sn GDE

**FIGURE 6:**
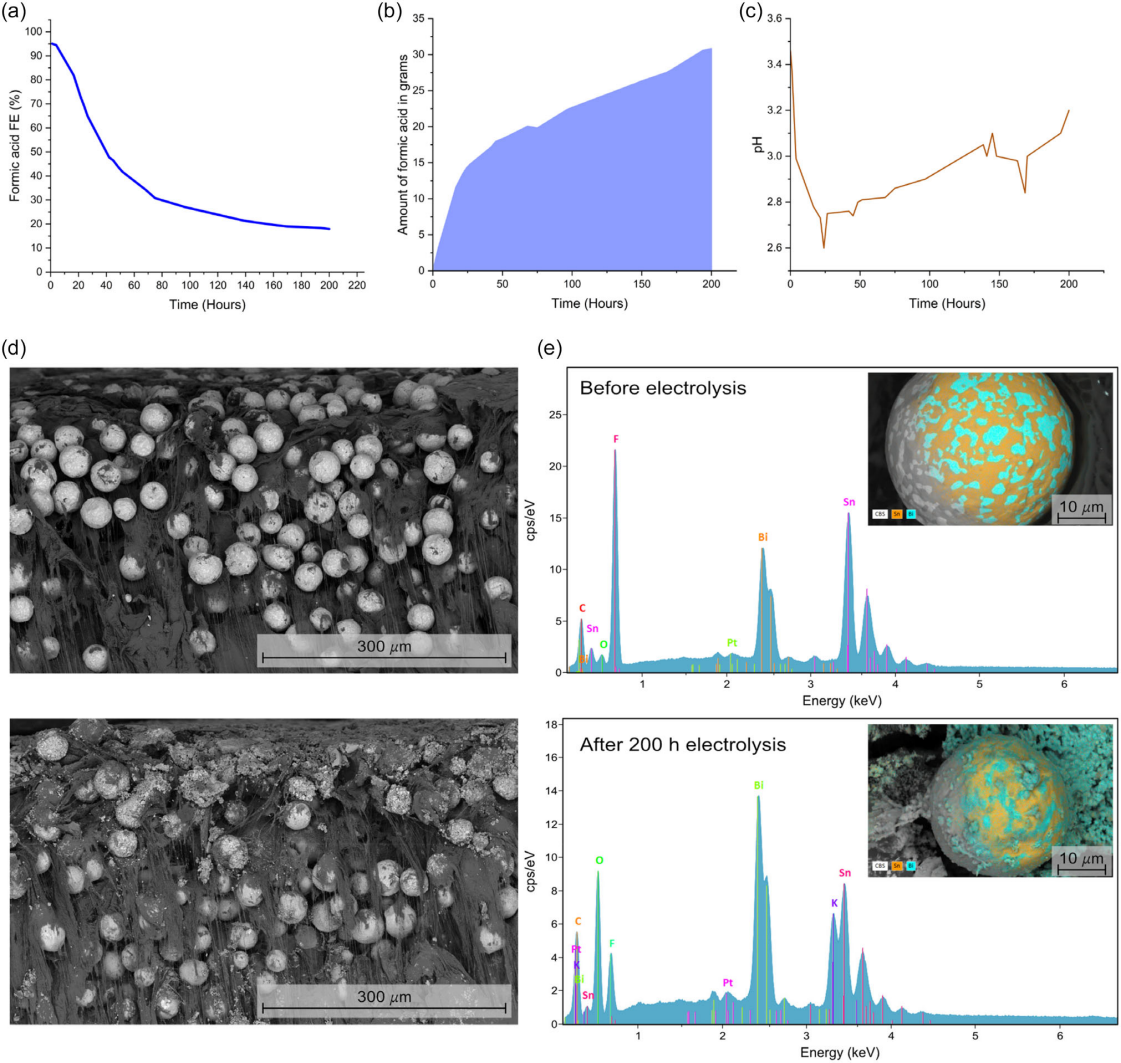
Long‐term eCO_2_R at the Bi_0.58_Sn_0.42_ GDE at –100 mA cm^−2^ with the catholyte initially at pH 3.5. (a) FE toward formic acid during 200 h. (b) Cumulative formic acid production in grams. (c) Catholyte pH evolution as a function of time. (d) Cross‐sectional SEM images of the GDE before electrolysis (top) and after 200 h of electrolysis (bottom). (e) EDX spectra and corresponding elemental maps (insets) of the catalyst particles before electrolysis (top) and after 200 h of electrolysis (bottom). The elemental maps show the phase‐segregated eutectic morphology, with Bi‐rich regions (cyan) and Sn‐rich regions (orange).

Given the mechanistic analysis that attributes selective formic acid formation to the eutectic's interfacial sites in its precatalyst state, as well as the morphological evolution observed for the Bi_0.58_Sn_0.42_ (Figure 4b), we assessed whether formic acid selectivity is preserved under extended operation (Figure 6). Long‐term electrolysis was conducted under the conditions that delivered the highest FE for formic acid (pH 3, –100 mA cm^−2^). To enhance buffering and accommodate gradual acidification from product accumulation, the catholyte was initially adjusted to pH 3.5.

As shown in Figure 6a, FE toward formic acid started at 95% and remained above 80% for the first 20 h, before gradually declining to ≈18% over 200 h. The total amount of formic acid accumulated in 2 L of catholyte was 3.8 g (Figure 6b), corresponding to a carbon conversion efficiency (CCE) of 4.5%, consistent with values reported for gas‐fed flow cells at comparable CO_2_ throughputs [36]. As demonstrated previously [37], reducing the CO_2_ flow rate by ≈50% can sustain Coulombic efficiencies while improving single‐pass carbon use.

The rate of formic acid production (slope of Figure 6b) correlates distinctively with the bulk pH evolution (Figure 6c). During the first 20 h, the high production rate (steep slope) coincides with a rapid drop in catholyte pH from 3.5 to 2.6, driven by the dissociation of the generated formic acid. Beyond 20 h, the production rate slows significantly, coincident with a gradual rise in pH. This shift marks the transition where the proton‐consuming HER begins to dominate, counteracting the acidification from the diminishing formate pathway. Consistent with observations in Figure 3b–d, HER becomes increasingly competitive once the bulk pH falls below 3, eventually stabilizing the pH near 3.

Nevertheless, the continued decline in FE to HCOOH despite partial pH recovery indicates that bulk pH drift alone cannot explain the performance loss. The sustained degradation suggests an irreversible catalyst evolution induced under acidic operating conditions, which likely reduces the density of active sites. To investigate the structural origin of this degradation, we performed post‐mortem characterization of the GDE. Cross‐sectional SEM imaging of the as‐prepared electrode (Figure 6d, top) confirms a uniform distribution of accessible spherical eutectic particles. In contrast, the cross‐section obtained after 200 h of operation (Figure 6d, bottom) reveals that these particles are buried under a overlayer. This observation clarifies the morphology change observed after 1 h (Figure 4b); the spherical particles are not degraded but rather encapsulated by this growth. Consequently, a significant fraction of the active Bi, Sn, and Bi–Sn interfacial sites becomes isolated from the bulk catholyte, leading to the observed loss of catalytic activity.

EDX spectra (Figure 6e) provide elemental insights into this surface evolution. The post‐electrolysis spectrum shows a marked increase in oxygen signal intensity relative to the metal peaks, alongside the emergence of a potassium signal. This suggests the formation of an oxide‐rich passivation layer and the precipitation of electrolyte‐derived potassium salts, which are absent in the as‐prepared GDE. Additionally, a reversal in metallic peak intensities is observed: while Sn signals dominate the fresh surface, Bi becomes the primary metallic component after operation. This trend is corroborated by the elemental maps (insets), which reveal a reduction in Sn‐rich regions (orange) on the spent electrode. This inversion is consistent with preferential Bi segregation to the surface, likely driven by surface energy minimization.

**FIGURE 7:**
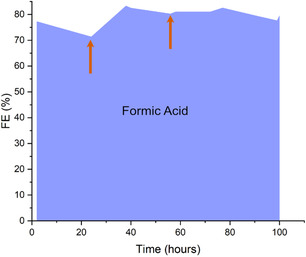
Performance of the Bi_0.58_Sn_0.42_ GDE with periodic catholyte replacement during long‐term operation at pH 3 and –100 mA cm^−2^. Orange arrows indicate the times at which the catholyte was replaced.

To mitigate this degradation, a subsequent durability test was performed at –100 mA cm^−2^ with periodic catholyte replacement to maintain pH 3 and limit product accumulation (Figure 7). Orange arrows in Figure 7 mark the times at which the catholyte was replaced. Under these conditions, the FE for formic acid remained markedly more stable, with a less than 10% FE decrease during 100 h of continuous operation. This improvement demonstrates that effective electrolyte management can alleviate acidification and proton depletion, thereby sustaining stable performance in acidic eCO_2_R systems.

The operational stability of the Bi_0.58_Sn_0.42_ GDE is notable when benchmarked against recent reports on acidic formic acid production (Table S2). While strategies such as protective coatings or cation‐assisted microenvironment engineering have been employed to mitigate HER by inducing locally alkaline conditions, the present system demonstrates that a simple eutectic architecture can achieve competitive longevity without complex surface passivation. These results suggest that the phase‐separated microstructure provides an intrinsically robust platform for operation in acidic media, enabling performance comparable to or exceeding that of several monometallic and doped analogues reported in the literature [38, 39, 40].

## 3 Conclusions

We prepared a eutectic Bi_0.58_Sn_0.42_ alloy catalyst for the electrochemical reduction of CO_2_ to formic acid under acidic conditions. The catalyst was systematically evaluated across different pH values and current densities, showing higher activity and selectivity than pure Bi and Sn electrodes. Scanning electron microscopy analysis confirmed a finely intermixed yet phase‐separated microstructure composed of Bi‐ and β‐Sn‐rich domains, without the formation of an intermetallic compound. Operation at pH 3 minimized hydrogen evolution while maintaining FEs above 80% for formic acid. Compared with the monometallic gas diffusion electrodes, the eutectic alloy clearly outperformed Bi and delivered a modest yet meaningful improvement over Sn. From a techno‐economic analysis perspective, this relative FE gain is relevant because the specific electricity consumption scales approximately with the inverse of FE; to first order, a ∼5% increase in FE therefore implies a ∼5% reduction in electricity demand per unit product [38, 41]. Future work should therefore assess the robustness of such engineered interfaces at current densities exceeding −100 mA cm^−2^ to fully exploit this synergistic effect in long‐term industrial operation. Although gradual catholyte basification and surface reconstruction led to performance decay, stable operation was readily restored through periodic electrolyte replacement, maintaining high faradaic efficiency over 100 h of continuous operation and underscoring the importance of electrolyte management in acidic CO_2_ electrolysis. Density functional theory calculations attributed the enhanced performance to a synergistic Bi–Sn interfacial effect. The binding energies of CO_2_‐to‐HCOOH intermediates lie between those of the monometallic counterparts, while *H adsorption under the high‐coverage conditions inherent to acidic media becomes energetically unfavorable. This non‐linear effect at the Bi–Sn interface suppresses hydrogen evolution by disfavoring *H accumulation and directs the reaction pathway toward CO_2_‐to‐HCOOH conversion. The resulting balance between weakened hydrogen binding and stabilized formate intermediates rationalizes the experimentally observed high selectivity for formic acid. Collectively, these results establish the Bi_0.58_Sn_0.42_ gas diffusion electrode as a robust and scalable platform for selective CO_2_ conversion in acidic media and highlight interfacial design as a promising route for the integration of CO_2_ electrolysis with renewable energy systems.

## 4 Experimental Section

Experiments were performed in a flow‐cell electrolyzer designed to facilitate scale‐up (Figure 2a). The reactor comprised catholyte and anolyte compartments separated by a bipolar membrane in reverse‐bias mode with the cation‐exchange layer (CEL) facing the cathode and the anion‐exchange layer (AEL) facing the anode. Water dissociation at the BPM interface generates protons (H^+^) and hydroxide ions (OH^–^), which migrate toward the cathode and anode, respectively, maintaining local acidity and alkalinity while stabilizing cell operation. The catholyte consisted of K_2_SO_4_ adjusted to pH 1, 2, or 3 with H_2_SO_4_, and the anolyte was 2 M KOH. Galvanostatic electrolysis was performed at current densities of –100, –200, –300, and –400 mA cm^−2^. Electrode performance was evaluated in terms of the FE for formic acid, cell potential (*V*_*cell*_), and working electrode potential (*E*_*we*_).

### 4.1 Electrode Fabrication

Bi, Sn, and Bi_0.58_Sn_0.42_ GDEs were prepared following a patented VITO process for the VITO core electrode [42]. The procedure involved separate preparation of the gas diffusion layer and the catalyst layer using a sequential stratified assembly technique, which were later consolidated into a single GDE unit (Figure 2c). The GDL was produced from a homogenized blend of ammonium bicarbonate (NH_4_HCO_3_, 98%, ACROS Organics) and PTFE (Dupont 669N X) at a mass ratio of 70:30 using a high‐shear mixer (Microtron MB 550, KINEMATICA AG, Switzerland), corresponding to 70 g of NH_4_HCO_3_ and 30 g of PTFE. In parallel, catalyst layers were prepared by mixing Bi (99.5%, 325 mesh size, Thermo Scientific Chemicals), Sn (99.8%, 325 mesh, Alfa Aesar), or Bi_0.58_Sn_0.42_ (58–42%, 325 mesh, American Elements) with NH_4_HCO_3_ and PTFE at a 70:20:10 mass ratio (metal:NH_4_HCO_3_:PTFE) using the same equipment.

Each blend was molded into a compact 10 cm × 10 cm square ‘cake’ using a 20‐ton hydraulic press (AC Hydraulics A/S). The cakes were individually calendared with precision‐engineered cold‐rolling metal rollers (custom‐built by VITO) to produce uniform sheets 0.5–0.6 mm thick. The final electrodes were obtained by compressing these two layers together in a secondary calendaring step. Finally, the integrated GDE was heat‐treated in a controlled‐environment oven (WTC Binder FD720) at 70°C for 6 h. This thermal step removed residual water and generated porosity via NH_4_HCO_3_ volatilization. The resulting metallic GDEs were carbon‐free and ready for eCO_2_R after activation.

### 4.2 Electrode Activation

Prior to CO_2_ electrolysis, electrodes were activated in a two‐electrode configuration to enhance conductivity and promote particle coalescence [43]. The GDE, mounted in a Ti current collector as the cathode, and a Pt‐coated Ta anode were placed in a gap‐flow cell without any membrane and 3 M KOH was used as electrolyte. A DC power supply (Elektro Automatik GmbH and Co. KG) applied a potential ramp from 5 V to 15 V in 10‐min increments, followed by a symmetric decrease. The current increased from ∼2 A at 5 V to 5 A at the same voltage at the end of the activation.

### 4.3 Electrode Characterization

Surface morphology and composition of the as‐prepared and post‐electrolysis electrodes were characterized using two complementary SEM instruments: a Phenom ProX (Thermo Fischer Scientific) equipped with long‐lifetime CeB_6_ electron source (Figures 4a,b, S2 and S3) and a Nova NanoSEM 450 (Thermo Fisher Scientific) coupled with a Bruker XFlash 6/60 EDS detector for cross‐sectional imaging and high‐resolution compositional analysis (Figure 6d,e).

XRD patterns were collected on an X’Pert Empyrean diffractometer (PANalytical) using Co‐Kα radiation (λ = 1.7890 Å) at 45 kV and 40 mA over a 2*θ* range of 0°–120° with a scan rate of 0.07° s^−1^.

### 4.4 Electrochemical Cell Configuration

The Micro Flow Cell (ElectroCell A/S; Figure 2b) was configured as a three‐compartment reactor (gas–catholyte–anolyte) separated by a Polyetheretherketone (PEEK)‐reinforced BPM (Fumasep FBM‐PK, reverse bias). The cathode consisted of the as‐prepared Bi–Sn or monometallic GDE mounted in a titanium holder, and a platinum‐coated tantalum plate served as the anode. A leak‐free Ag/AgCl reference electrode was positioned on the cathode side to monitor the working electrode potential. Ethylene–propylene–diene monomer (EPDM) rubber‐based gaskets and PTFE flow frames ensured sealing and uniform distribution of gas and liquid streams.

### 4.5 Electrochemical CO_2_ Reduction

The catholyte (0.25 M K_2_SO_4_, acidified to pH 1–3 with H_2_SO_4_) and anolyte (2 M KOH) were circulated from 250 mL reservoirs at 40 mL min^−1^ using peristaltic pumps. CO_2_ was continuously supplied at 30 mL min^−1^ (ambient pressure, room temperature) to the cathode gas side through the GDL in flow‐through mode. Durability tests employed 2 L of catholyte and 1 L of anolyte, with continuous monitoring of catholyte pH. Liquid samples were periodically collected to track the evolution of liquid‐phase reaction products.

The catholyte pH and conductivity were measured using a pH probe (Hach HQ 2200 portable meter with combined pH electrode) and a conductivity meter (Hach HQ 2200 portable meter with combined conductivity cell) inserted directly into the catholyte reservoir bottle. Both probes were calibrated prior to each experiment with commercial buffer/standard solutions (Singlet pH buffer solution and standard solution, HACH LANGE GmbH).

Gaseous products were quantified using gas chromatography (GAS Compact GC 4.0, Global Analyser Solutions) equipped with two thermal conductivity detectors (TCDs) for H_2_ and CO and a flame ionization detector (FID) for CH_4_, C_2_H_6_, and C_2_H_4_. Helium served as the carrier gas for the TCDs (1 mL min^−1^), and air was used for the FID (300 mL min^−1^).

At the end of electrolysis, 4 mL aliquots of both catholyte and anolyte were collected for liquid‐phase analysis by high‐performance liquid chromatography (Agilent 1200 Series, Agilent Technologies) and gas chromatography–mass spectrometry with headspace injection (Trace‐GC–MS with HS, Thermo Fisher Scientific). The electrolyzer setup used for all electrodes is shown in Figure S1. Details of FE calculations are provided in the Supporting Information.

### 4.6 DFT Calculations

Periodic DFT calculations were carried out using the Vienna Ab Initio Simulation Package (VASP, version 6.4.2) [44], adopting the Bayesian error estimation functional with van der Waals correlation (BEEF‐vdW) [45]. Core and valence electrons were represented by projector‐augmented wave (PAW) pseudopotentials [46] and plane waves with a kinetic energy cutoff of 500 eV, respectively. Electronic occupancies were treated using a Gaussian smearing of 0.1 eV for gas‐phase species, while a first‐order Methfessel–Paxton smearing of 0.1 eV was applied to slab models; all calculations were spin polarized. Molecular species were optimized at the Γ‐point, whereas periodic slabs were sampled using a Γ‐centered k‐point mesh with a spacing of ≃ 0.11 Å^−1^, determined from convergence tests ensuring the total electronic energy varied by less than 2 meV per atom (Figure S6). Geometry relaxations were performed with a conjugate‐gradient algorithm with a step size of 0.1 Å and convergence criteria of 10^−6^ eV and 0.01 Å eV^−1^ for electronic and ionic steps, respectively.

Gibbs energy corrections were computed at the experimental temperature of 298 K and 1 atm of pressure using the Atomic Simulation Environment (ASE) [47] thermochemistry module. Gas‐phase species were treated in the ideal gas approximation, while adsorbates were modeled in the harmonic limit. In these calculations, all atoms were vibrated in molecular systems, whereas only adsorbates were vibrated in slab models. Vibrational frequencies were obtained by finite differences with a 0.02 Å step size.

### 4.7 Bulk Structure Optimization

All structures were generated using the Python Materials Genomics (pymatgen) package [48]. A 15 Å vacuum gap was applied in all directions for molecular calculations to avoid spurious interactions between periodic images. For bulk Sn and Bi, experimentally observed crystal structures were adopted: tetragonal Sn (I4_1_/amd, mp‐84) and trigonal Bi (R‐3 m, mp‐23 152) from the Materials Project database [49]. The lattice parameters were varied by ±1% in five increments, and each structure was relaxed with atomic positions optimized at fixed cell volume. The resulting energy–volume data were fitted to the Birch–Murnaghan equation of state [50], and the minimum was subsequently relaxed at constant volume while allowing cell–shape optimization to obtain the equilibrium bulk geometries.

### 4.8 Surface Slabs Generation

For each (*hkl*) facet, slab models of at least four interplanar spacings ($4d_{hkl}$) were constructed and separated by 20 Å of vacuum along the surface normal. Because a single crystallographic plane may include multiple atomic layers, the total slab thickness sometimes exceeded four atomic layers. The surface energy (γ) was computed as:

(2) $$\gamma =\frac{E_{\text{slab}}-nE_{\text{bulk}}}{2A}$$

where $E_{\text{slab}}$ and $E_{\text{bulk}}$ are the total energies of the slab and bulk energy per atom, respectively, n is the number of atoms in the slab, and A is the surface area; the factor of 2 accounts for the two terminations of the surface slab model. Convergence with respect to slab thickness was verified for the most stable terminations. Slabs were centered in the simulation cell, with atoms in the lower half fixed at bulk positions and the upper half fully relaxed. Comparison with fully relaxed slabs confirmed negligible energy differences (≤0.02 meV atom^−1^ for Sn(200), 8 × 10^−4^ meV atom^−1^ for Bi(012)). Computed surface energies, Wulff constructions, and thickness–convergence tests are provided in the Supporting Information, together with details of the Bi_0.58_Sn_0.42_ in‐plane heterostructure model.

### 4.9 Surface Coverages

Hydrogen coverage studies were performed on *p*(1 × 1) − Sn(200), *p*(2 × 1) − Sn(200), and *p*(2 × 2) − Bi(012) slabs. A single *H atom was first placed at each symmetrically distinct adsorption site to determine the preferred site. Additional *H atoms were then added sequentially, enumerating all unique configurations until one of the following criteria was met: (i) one monolayer (IUPAC definition) was reached; (ii) an adsorbate migrated to a previous sampled site; or (iii) hydrogen evolution occurred. The resulting coverage resolutions were 1/2 ML for *p*(1 × 1) − Sn(200), 1/4 ML for *p*(2 × 1) − Sn(200), and 1/6 ML for *p*(2 × 2) − Bi(012), enabling refined surface–phase diagrams. For example, *p*(1 × 1) − Sn(200) allows sampling of the 0.5 ML and 1 ML lines whereas the *p*(2 × 1) − Sn(200) slab for 0.25 ML and 0.75 ML.

The relative Gibbs energies of the *H‐covered surfaces ($\Delta G_{nH}$) were evaluated within the computational hydrogen electrode (CHE) model [51]:

(3) $$\Delta g_{\mathrm{nH}}\left(0V_{\mathrm{RHE}}\right)=\frac{G_{\mathrm{nH}}-\left(G_{*}+\frac{n}{2}G_{H_{2}}\right)}{N_{\text{surf}}}$$

where $G_{\mathrm{nH}}$ is the Gibbs energy of a slab with n adsorbed *H atoms, $G_{*}$ is the potential energy of the bare slab, $G_{H_{2}}$ is the Gibbs energy of a gas‐phase H_2_, and $N_{\text{surf}}$ is the number of surface atoms. Dividing by $N_{\text{surf}}$ normalizes $\Delta g_{\mathrm{nH}}$ per surface site, ensuring the direct comparison between supercells of different size. The computed values are summarized in Table S5.

Surface coverage diagrams for pure Sn and Bi were obtained by plotting $\Delta g_{\mathrm{nH}}$ as a function of potential:

(4) $$\Delta g_{\mathrm{nH}}\left(U_{\mathrm{RHE}}\right)=\Delta g_{\mathrm{nH}}+\theta U_{\mathrm{RHE}}$$

where $\theta =n/N_{\text{surf}}$ is the surface coverage and $U_{\mathrm{RHE}}$ is the potential vs RHE, converted from Ag/AgCl according to:

(5) $$U_{\mathrm{RHE}}=U_{\mathrm{Ag}/\text{AgCl}}+0.197+0.059\;\mathrm{pH}$$

with 0.197 V being the standard potential of Ag/AgCl (sat. KCl) vs the standard hydrogen electrode (SHE) at 25°C.

For HCOO* and *COOH adsorption on the bare surfaces, all symmetrically distinct surface sites and rotational orientations were enumerated to identify unique configurations. On *p*(2 × 2) − Sn(200), five inequivalent sites combined with three rotational orientations (0°, 45°, 90°) yielded 15 configurations per intermediate (30 total). On *p*(2 × 2) − Bi(012), five sites with two orientations (0°, 90°) produced 10 configurations per intermediate (20 total). All configurations were fully relaxed.

For adsorption on H‐covered surfaces, one H atom was removed from an *ontop* site to create a vacancy, which was then occupied by the intermediate in its most stable orientation from the bare surface calculations. For Bi_0.58_Sn_0.42_, a hydrogen coverage of 1 ML was assumed at −1.70 V_RHE_, consistent with the calculated resting states of Bi(012) and Sn(200). The alloy was modeled as a lateral heterostructure between Sn(200) and Bi(012), and intermediates were placed on the Bi domain, the Sn domain, or at the Bi–Sn interfacial domain. HCOO*, which binds in a bidentate mode, was considered in all three regions, whereas *COOH (binding through C) was evaluated on the Bi and Sn domains. Each configuration was fully optimized together with the H‐covered surface containing one vacancy.

Adsorption energies of HCOO* and *COOH were modeled as PCET steps using the CHE formalism:

(6) $$\Delta G_{\text{*COOH}/(n-1)H}=G_{\text{*COOH}/(n-1)H}-\left(G_{(n-1)H}+G_{{\mathrm{CO}}_{2}}+\frac{1}{2}G_{H_{2}}\right)$$

(7) $$\Delta G_{\text{HCOO*}/(n-1)H}=G_{\text{HCOO*}/(n-1)H}-\left(G_{(n-1)H}+G_{{\mathrm{CO}}_{2}}+\frac{1}{2}G_{H_{2}}\right)$$

where $G_{\text{*COOH}/(n-1)H}$ and $G_{\text{HCOO*}/(n-1)H}$ are the Gibbs energies of the intermediates on the hydrogenated surfaces with one vacancy, $G_{{\mathrm{CO}}_{2}}$ and $G_{H_{2}}$ are those of gas‐phase CO_2_ and H_2_, and $G_{(n-1)H}$ is that of the H‐covered surface after hydrogen removal.

## Supporting Information

Additional supporting information can be found online in the Supporting Information section.

Supporting information for this article is given via a link at the end of the document.

The supporting information is attached. Supporting Fig. S1: Visual representation of experimental setup used for eCO_2_R. Supporting Fig. S2: SEM images of the Bi_0.58_Sn_0.42_ GDE acquired at varying magnifications, illustrating the surface morphology before (a, b) and after (c, d) electrolysis. × : SEM images of monometallic Sn GDEs (a, b) and Bi GDEs (c, d), before (a, c) and after (b, d) electrolysis. Supporting Fig. S4: XRD pattern of the Bi_0.58_Sn_0.42_ GDE (a) before and (b) after eCO_2_R, in which Bi‐related reflections are marked by circles and Sn‐related reflections by triangles. Supporting Fig. S5: Cell voltage (*V*_*cell*_) and working electrode potential (*E*_*we*_) of the Bi_0.58_Sn_0.42_ GDE during eCO_2_R at (a) pH 1, (b) pH 2, and (c) pH 3 across current densities of 100–400 mA cm^–2^. Supporting Fig. S6: Convergence of total energy with respect to k‐point sampling for bulk (a) Sn and (b) Bi. The absolute energy difference per atom is plotted as a function of the Monkhorst–Pack k‐point grid, referenced to the densest mesh (23 × 23 × 43 for Sn and 35 × 35 × 11 for Bi). The blue shaded region indicates the 2 meV atom^–1^ convergence threshold. Supporting Fig. S7: Wulff constructions of the equilibrium crystal morphologies for (a) Sn and (b) Bi. Supporting Fig. S8: Convergence of surface energy (Δγ) with respect to slab thickness for (a) Sn(200) and (b) Bi(012). Energies are referenced to the thickest slab considered (Δγ = 0). The blue shaded region denotes the estimated thermal noise normalized by the slab area. Supporting Fig. S9: Schematic illustration of the construction of the in‐plane Bi–Sn heterostructure slab. (a) Optimized Bi(012) (pink) and Sn(200) (blue) slabs are concatenated along the y‐direction, introducing a lattice mismatch, ε. Here, a and b denote the in‐plane lattice parameters, while c corresponds to the out‐of‐plane parameter defining the slab vacuum. (b) Integer multiples are applied to minimize the mismatch and form commensurate supercells. The example shown corresponds to commensuration along x and concatenation along y. (c) The commensurate Bi and Sn supercells are then joined laterally to produce the final Bi–Sn heterostructure. Supporting Table S1: Lattice parameters and relative lattice changes of the Bi β‐Sn phases within the eutectic Bi0.58Sn0.42 GDE before and after electrolysis. The percentage change is calculated as $(P_{after}-P_{before})/P_{before}\times 100$, where P represents the lattice parameter *a* = *b* or *c.* Supporting Table S2: Comparison of eCO_2_R performance of previously reported high‐performance Bi/Sn catalysts in the literature. Entries are sorted by publication year. Supporting Table S3: Computed surface energies (γ, in eV Å^–2^) for Sn and Bi facets. Supporting Table S4: Relative lattice mismatch for all Bi(012)–Sn(200) in‐plane vector matchings. The best integer multiples (m,n) of the Bi and Sn unit vectors and the resulting mismatches are listed. Supporting Table S5: Summary of *H adsorption Gibbs energies per surface atom (in eV) at 0 V_RHE_ ($\Delta g_{\mathrm{nH}}(0V_{\mathrm{RHE}})$) on Sn and Bi surfaces. θ denotes surface coverage (ML), n the number of adsorbed H atoms, and $N_{surf}$ the number of surface atoms in the supercell. ‘HER’ indicates that hydrogen is evolved during geometry relaxation, marking the coverage limit.

## Author Contributions

**Avni Guruji**: conceptualization (lead), formal analysis (equal), investigation (lead), visualization (equal), writing – original draft (lead). **Alejandro Cañete‐Arché**: conceptualization (equal), formal analysis (equal), investigation (lead), visualization (lead), writing – original draft (lead). **Yuvraj Birdja**: investigation (equal), writing – review & editing (supporting). **Ranjith Prasannachandran**: Investigation (equal), Writing – review & editing (supporting). **Max García‐Melchor**: conceptualization (equal), funding acquisition (lead), resources (lead), supervision (lead), validation (equal), writing – review & editing (equal). **Deepak Pant**: conceptualization (lead), funding acquisition (lead), resources (lead), supervision (lead), validation (equal), writing – review & editing (equal).

## Supporting information

Supplementary Material
