# Supplementary material for: Electrochemical CO2 Reduction on a Bi–Sn Eutectic Alloy in Acidic Media for Formic Acid Production
Source: ChemSusChem. 2026 Apr 21;19(8):e202502541. doi: 10.1002/cssc.202502541 (PMC13099273; doi:10.1002/cssc.202502541)
Supplement: Supplementary file 1 — Supplementary Material [file CSSC-19-e202502541-s001.pdf]

Supporting Information

Electrochemical CO_2 Reduction on a Bi–Sn Eutectic Alloy in Acidic Media for Formic Acid Production

---

Avni Guruji†[a][b], Alejandro Cañete-Arché†[c], Yuvraj Y. Birdja[a], Ranjith Prasannachandran[a], Max García-Melchor*[c][d][e], Deepak Pant*[a][f]

[a] Avni Guruji, Yuvraj Y. Birdja, Ranjith Prasannachandran, Deepak Pant
Electrochemistry Excellence Centre (ELEC), Materials & Chemistry Unit
Flemish Institute for Technological Research (VITO)
Boeretang 200, Mol 2400, Belgium
E-mail: deepak.pant@vito.be

[b] Avni Guruji
Dipartimento di Scienza Applicata e Tecnologia,
Politecnico di Torino, 10129 Torino, Italy

[c] Alejandro Cañete-Arché, Prof. Max García-Melchor
School of Chemistry, Trinity College Dublin,
College Green, Dublin 2, Ireland

[d] Prof. Max García-Melchor
Center for Cooperative Research on Alternative Energy (CIC energiGUNE), Basque Research and Technology Alliance (BRTA), Alava Technology Park,
Albert Einstein 48, 01510 Vitoria-Gasteiz, Spain
E-mail: maxgarcia@cicenergigune.com

[e] Prof. Max García-Melchor
IKERBASQUE, Basque Foundation for Science,
Plaza de Euskadi 5, 48009 Bilbao, Spain

[f] Deepak Pant
Center for Advanced Process Technology for Urban Resource Recovery (CAPTURE),
Frieda Saeysstraat 1, Zwijnaarde, 9052 Belgium

† Equal Contribution

Table of Contents

Experimental Section **3**

Electrochemical CO_2 Reduction Setup 3

Calculation of Faradaic Efficiency 3

SEM Characterization **4**

X-Ray Diffraction of $Bi_{0.58}Sn_{0.42}$ GDE **5**

Cell Voltage and Working Electrode Potential **5**

Computational Details **6**

Convergence Tests for Bulk Bi and Sn 6

Bulk Structure Optimization 6

Surface Slabs Generation and Surface Energy Calculations 6

Construction of Bi–Sn Heterostructure Slab 8

Adsorption Energies of *H on Bi and Sn Surfaces 9

Experimental Section

Electrochemical CO_2 Reduction Setup

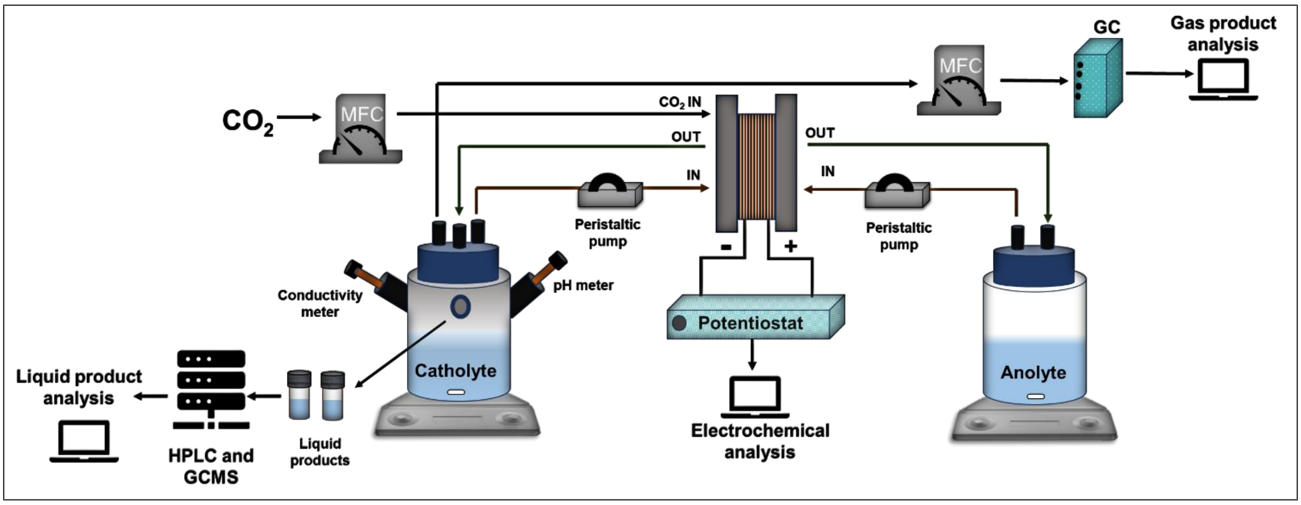

Figure S1. Visual representation of experimental setup used for eCO_2R.

Calculation of Faradaic Efficiency (FE)

$$FE(\%) = \frac{Q_{Experimental}}{Q_{Theoretical}} \times 100 \tag{S1}$$

For liquid products:

$$Q_{Experimental} = \frac{C \times V \times F \times n}{M} \tag{S2}$$

$$Q_{Theoretical} = I \times t \tag{S3}$$

Where C is the concentration of liquid product, V is the volume of electrolyte, n is the number of electrons, F is the Faraday constant (96485 C mol^{-1}), M is the molar mass of product, I is the applied current, and t is the time.

For gaseous products:

$$FE(\%) = \frac{f \times C \times F \times n}{V_m \times I} \times 100 \tag{S4}$$

Where f is the flow rate of the outlet gas, C is the concentration of gaseous product, V_m is the molar volume of gas, and I is the applied current.

SEM Characterization

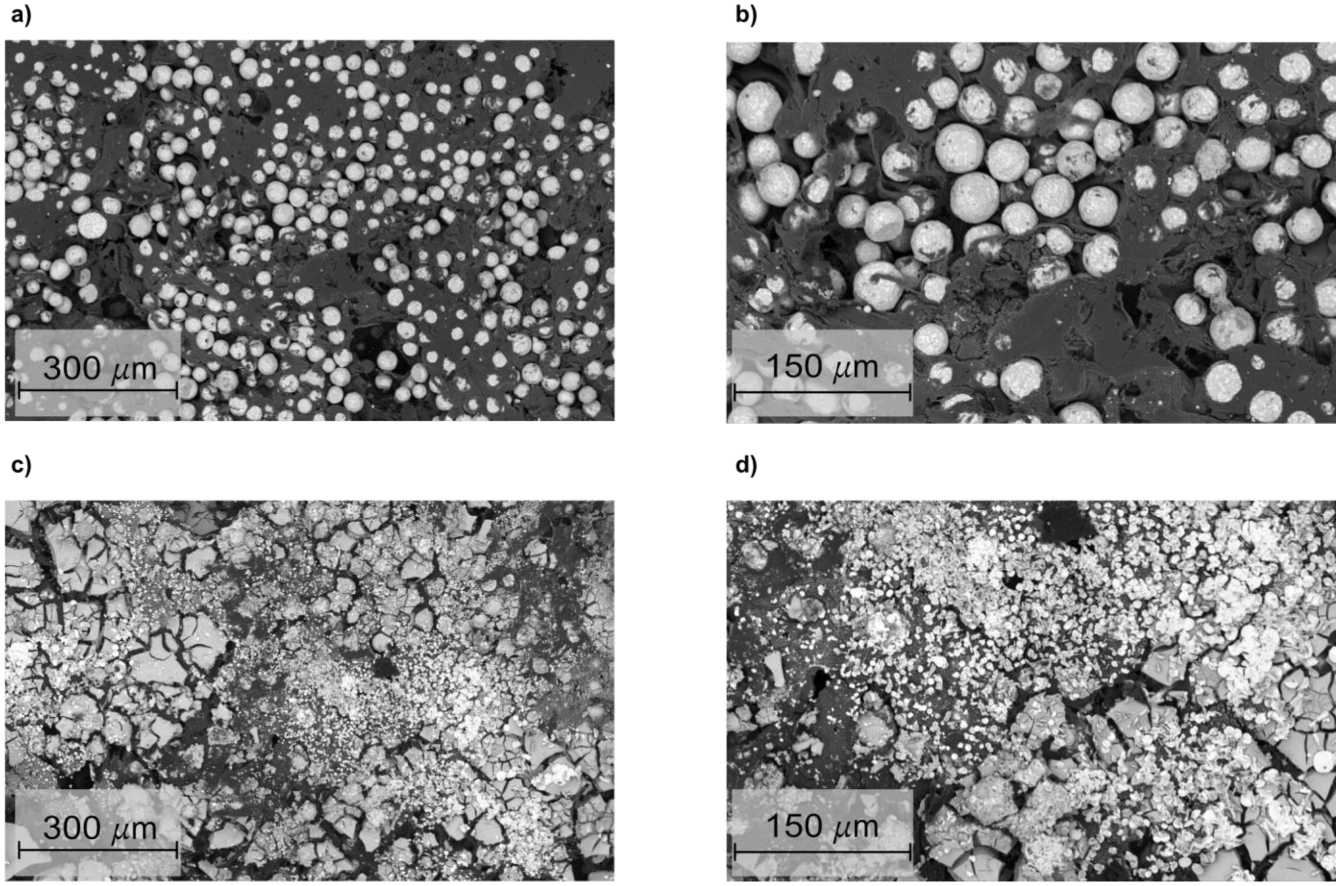

Figure S2. SEM images of the $Bi_{0.58}Sn_{0.42}$ GDE acquired at varying magnifications, illustrating the surface morphology before (a, b) and after (c, d) electrolysis.

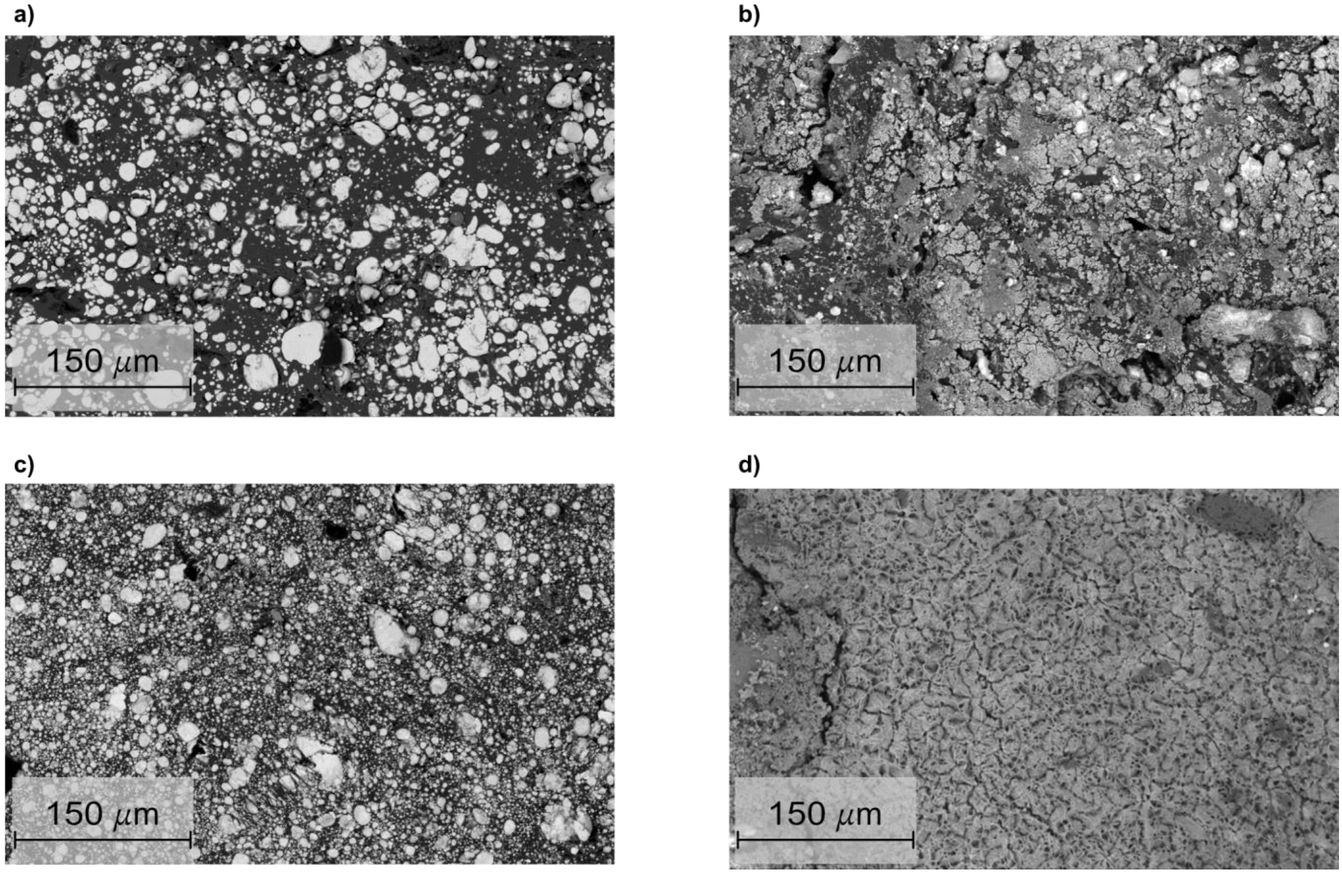

Figure S3. SEM images of monometallic Sn GDEs (a, b) and Bi GDEs (c, d), before (a, c) and after (b, d) electrolysis.

X-Ray Diffraction of the $Bi_{0.58}Sn_{0.42}$ GDE

The resulting XRD patterns of both before and after electrolysis Bi–Sn GDEs are shown in **Figure S4**. Distinct diffraction peaks corresponding to Bi and Sn phases were identified, with Bi-related reflections marked by circles and Sn-related reflections by triangles. A peak attributed to PTFE (polytetrafluoroethylene) was also observed, confirming the presence of the hydrophobic binder in the GDE structure.

Bi phase was identified using the JCPDS card no. 85-1329 (rhombohedral Bi), with dominant reflections at 2θ ≈ 31.65° [(012) plane] and 46.35° [(110) plane]. β-Sn phase was confirmed using the JCPDS card no. 04-0673 (tetragonal Sn), showing peaks at 2θ ≈ 35.76° [(200) plane] and 37.41° [(101) plane].

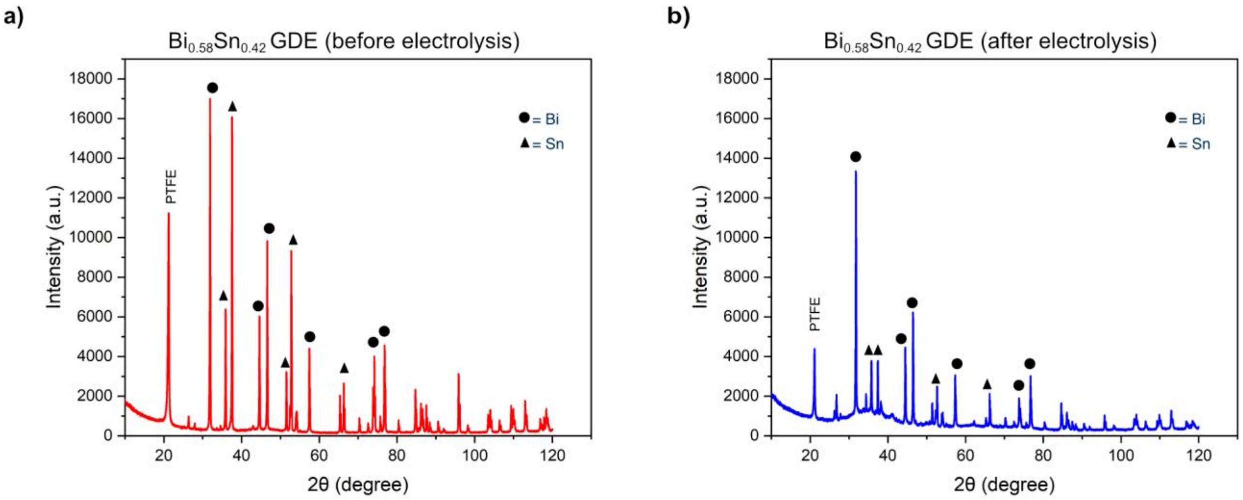

Figure S4. XRD pattern of the $Bi_{0.58}Sn_{0.42}$ GDE (a) before and (b) after eCO_2R, in which Bi-related reflections are marked by circles and Sn-related reflections by triangles.

The lattice parameters for both phases were calculated before and after electrolysis (**Table S1**). The β-Sn phase exhibited a uniform expansion, with the lattice constants increasing from *a* = *b* = 5.8184 Å and *c* = 3.1729 Å to *a* = *b* = 5.8326 Å (+0.24%) and *c* = 3.1821 Å (+0.29%), respectively. In contrast, the Bi phase showed anisotropic lattice distortion, where the lattice constants shifted from *a* = *b* = 4.5500 Å and *c* = 11.850 Å to *a* = *b* = 4.5350 Å (–0.33%) and *c* = 11.870 Å (+0.17%), respectively.

Table S1. Lattice parameters and relative lattice changes of the Bi β-Sn phases within the eutectic Bi0.58Sn0.42 GDE before and after electrolysis. The percentage change is calculated as $(P_{after} - P_{before})/P_{before} \times 100$, where P represents the lattice parameter *a* = *b* or *c.*

Phase	Parameter	Before	After	Change (%)
Bi	*a*	4.550	4.535	–0.33
	c	11.850	11.870	+0.17
β-Sn	*a*	5.8184	5.8326	+0.24
	c	3.1729	3.1821	+0.29

Cell Voltage and Working Electrode Potential

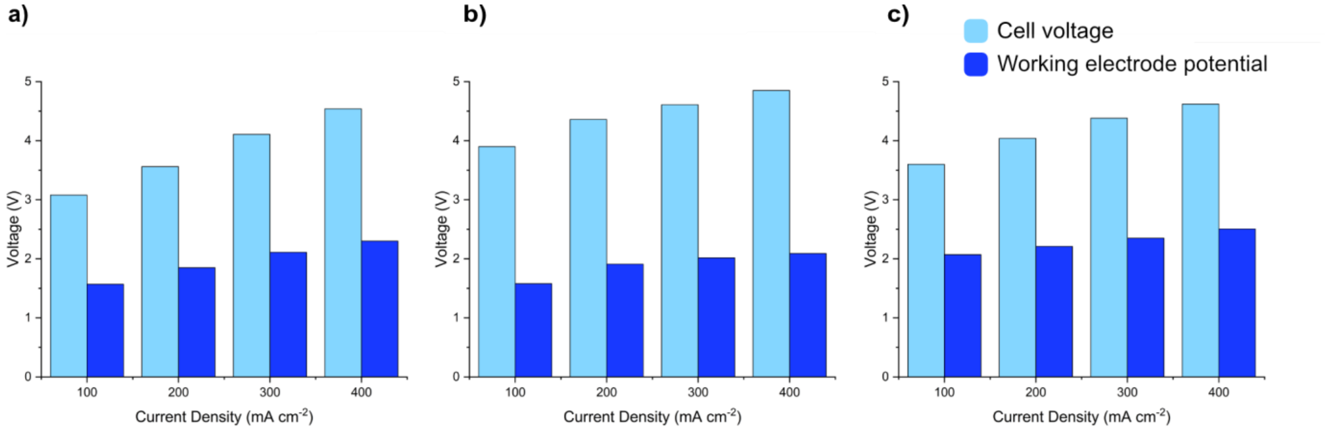

Figure S5. Cell voltage (V_{cell}) and working electrode potential (E_{we}) of the $Bi_{0.58}Sn_{0.42}$ GDE during eCO_2R at (a) pH 1, (b) pH 2, and (c) pH 3 across current densities of 100–400 mA cm^{-2}.

eCO_2R Studies Benchmark

Table S2. Comparison of eCO_2R performance of previously reported high-performance Bi/Sn catalysts in the literature. Entries are sorted by publication year.

Catalyst	Electrolyte	Cell type	Product	Current density (mA cm^{-2})	FE (%, vs RHE)	Stability (h)	Ref.
**Bi nanoparticles on Sn nanosheets**	0.5 M $KHCO_3$ (pH 7.2)	H-cell	Formate	56 (formate)	94 ± 2% at –1.14 V	100 h	Wen et al. (2018)[1]
**Bi–Sn eutectic nano-alloy**	0.1 M $KHCO_3$ (pH 6.8)	H-cell	Formate	8.5 (formate)	78% at –1.10 V	1 h	Tang et al. (2019)[2]
**Bi-doped SnO nanosheets**	0.1 M $KHCO_3$ (pH 6.8)	H-cell	Formate	12 (total)	93% at –1.70 (vs Ag/AgCl)	30 h	An et al. (2019)[3]
**Bi-doped SnO_x nanoshells**	0.5 M $KHCO_3$	Flow cell	Formate	20.9 (formate)	95.8% at –0.88 V	50 h	Yang et al. (2020)[4]
**Sn–Bi/SnO_2**	1.0 M $KHCO_3$ (pH 11)	Flow cell	Formate	100, 200, 300 (total)	95% at ~ –1.0 V	2400 h	Li et al. (2021)[5]
**SnO_2 nanoparticles on Bi2O_3 sheets**	0.1 M $KHCO_3$ (pH 6.8)	H-cell	Formate	~3.5 (total)	~80% at –1.0 V	12 h	Tian et al. (2021)[6]
**Sn-doped Bi/BiO_x nanowires**	1.0 M KOH (pH 14)	Flow cell	Formate	~100 (formate)	~100% at –0.7 V	20 h	Zhao et al. (2021)[7]
**Sn–Bi bimetallic interface**	0.5 M $KHCO_3$	Flow cell	Formate	~320 (total)	96.4 ± 2.5% at –0.84 V	160 h	Ren et al. (2022)[8]
**K^+-assisted Bi nanosheets**	0.05 M H_2SO_4 + 3M KCl (pH < 1)	Flow cell	**Formic acid**	237.1 (total)	92.2% at –1.23 V	8 h	Qiao et al. (2022)[9]
**$Cs_3Bi_2Br_9$/C**	3mM HBr + 0.5 M CsBr (pH 2.5)	H-cell	**Formic acid**	133.7 (formic acid)	92% at –0.95 V	20h	Wang et a. (2022)[10]
**Reservoir Bi nanosheets**	0.05 M H_2SO + 0.5 M $KHCO_3$ (pH 2)	Flow cell	**Formic acid**	200 (total)	>90% at ~ –1.8 V	45 h	Chi et al. (2023)[11]
**Si–C/BiSn (5–10 wt%)**	0.5 M H_2SO_4 (pH 1)	Flow cell	**Formic acid**	100 (total)	>90% at –1.5 V	120 h	Li et al. (2023)[12]
**S-doped Sn**	0.5 M K_2SO_4 (pH 3)	Flow cell	**Formic acid**	400 (total)	85% at –2V	13.5 h	Shen et al. (2023)[13]
**Bi–Sn eutectic ($Bi_{0.58}Sn_{0.42}$)**	0.25 M K_2SO_4 + H_2SO_4 (pH 3)	Flow cell	**Formic acid**	100 (total)	81.26 ± 3.06% at –1.70 V	100 h	**This work**

Computational Details

Convergence Tests for Bulk Bi and Sn

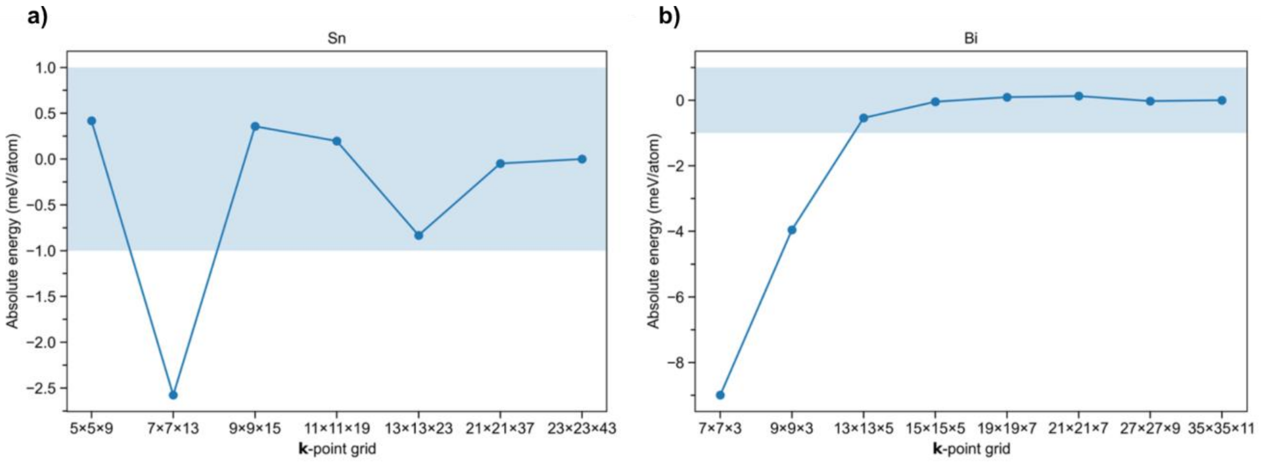

Figure S6. Convergence of total energy with respect to k-point sampling for bulk (a) Sn and (b) Bi. The absolute energy difference per atom is plotted as a function of the Monkhorst–Pack k-point grid, referenced to the densest mesh (23×23×43 for Sn and 35×35×11 for Bi). The blue shaded region indicates the 2 meV atom^{-1} convergence threshold.

Bulk Structure Optimization

The experimentally measured lattice parameters for Sn ($a = b = 5.8256$ Å, $c = 3.1774$ Å) show excellent agreement with the theoretical values ($a = b = 5.9119$ Å, $c = 3.1645$ Å), corresponding to deviations of ~1.46% for the *a*/*b* parameters and 0.41% for *c*. Similarly, for Bi, the experimental parameters ($a = b = 4.5460$ Å, $c = 11.8620$ Å) are consistent with theoretical predictions ($a = b = 4.5263$ Å, $c = 12.3724$ Å), with minimal deviation in the basal plane (0.44% deviation) and reasonable agreement along the *c*-axis (~4.1%).

Surface Slabs Generation and Surface Energy Calculations

Table S3 summarizes the computed surface energies for all facets identified in the XRD patterns of the Sn and Bi electrodes. These values were used for Wulff constructions of equilibrium crystal morphologies (**Figure S7**). The calculated surface energies for Bi facets (0.021–0.038 eV Å^{-2}) agree well with isotropic averages extrapolated from experimental surface tension data (~0.027 eV Å^{-2}).[14] In contrast, surface energies for selected ß-Sn facets (0.025–0.033 eV Å^{-2}) are slightly lower than the experimental isotropic average (~0.043 eV Å^{-2}).[14] Convergence with respect to slab thickness was verified for the most stable surfaces, Sn(200) and Bi(012), both of which converged at six atomic layers (**Figure S8**).

For the $Bi_{0.58}Sn_{0.42}$ eutectic alloy, it is well established that the Bi–Sn binary system does not form intermetallic compounds and that phase separation persists even at the nanoscale.[2] SEM imaging of the eutectic alloy revealed lamellar Bi- and ß-Sn-rich domains, consistent with literature reports describing the eutectic microstructure as coexisting Bi and Sn solids. Accordingly, the Bi–Sn surface was modeled as an in-plane heterostructure interface, where Bi and Sn slabs form lateral boundaries.

To construct the $Bi_{0.58}Sn_{0.42}$ heterostructure, we used the optimized six-layer slabs of Bi(012) and Sn(200) and evaluated all possible of in-plane matchings orientations: (i) $x_{\mathrm{Bi}} \leftrightarrow x_{\mathrm{Sn}}$, (ii) $y_{\mathrm{Bi}} \leftrightarrow y_{\mathrm{Sn}}$, (iii) $x_{\mathrm{Bi}} \leftrightarrow y_{\mathrm{Sn}}$, and (iv) $y_{\mathrm{Bi}} \leftrightarrow x_{\mathrm{Sn}}$ (**Figure S9**). For each pairing, integer multiples (m,n) were scanned to minimize the relative lattice mismatch:

$$\epsilon = \frac{|mL_{\mathrm{Sn}} - nL_{\mathrm{Bi}}|}{\frac{1}{2}(mL_{\mathrm{Sn}} + nL_{\mathrm{Bi}})} \qquad (S5)$$

where L_{Sn} and L_{Bi} denote the lattice parameters of Sn and Bi, respectively, along the matched direction. **Table S4** lists the lowest-strain combinations, all yielding mismatches below 2%, indicating that coherent interfaces are feasible. The best match, $x_{\mathrm{Bi}} \leftrightarrow x_{\mathrm{Sn}}$ with $m = 10$, $n = 7$, gives a mismatch of only 0.12% and was selected for the representative $Bi_{0.58}Sn_{0.42}$ slab model.

The Bi slab was vertically shifted so that its geometric mid-plane coincided with that of Sn, ensuring coplanarity prior to lateral stitching. The resulting *p*(10×1)-Sn(200) and p(7×2)-Bi(012) supercells were concatenated along y to form the Bi–Sn interface, reproducing the experimental Bi:Sn ratio of 58:42. **Figures S9** illustrates this construction, and **Figures 4c** and **4d** show the final structure generated. The shorter Sn supercell ($10a_{\mathrm{Sn}}$) defines the common length, imposing a slight compressive strain on the Bi domain; all atomic positions were subsequently relaxed. Constraining the bottom half of the slab to bulk positions produced negligible energy differences (0.02 meV atom^{-1}) relative to fully relaxed structures.

Table S3. Computed surface energies (γ, in eV Å^{-2}) for Sn and Bi facets.

**Metal**	**Facet (*hkl*)**	γ
Sn	(200)	0.0248
	(101)	0.0273
	(301)	0.0277
	(211)	0.0322
	(220)	0.0333
Bi	(012)	0.0208
	(110)	0.0237
	(104)	0.0322
	(202)	0.0376

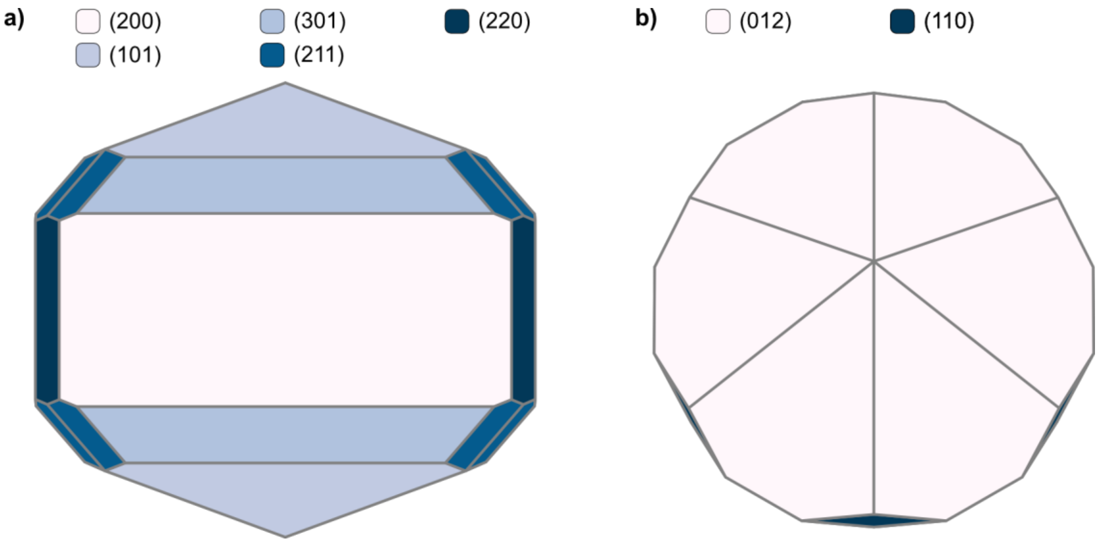

Figure S7. Wulff constructions of the equilibrium crystal morphologies for (a) Sn and (b) Bi.

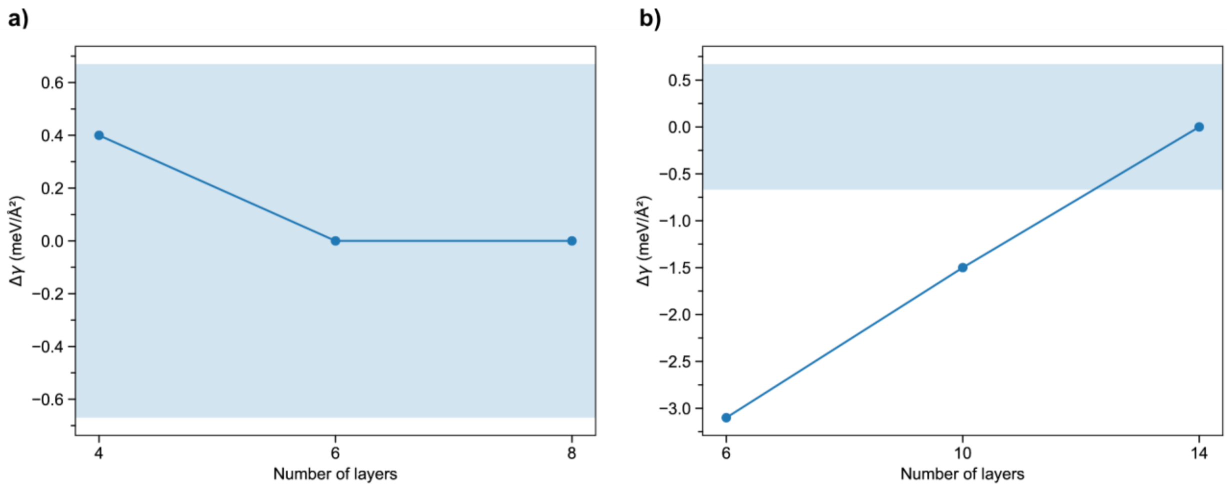

Figure S8. Convergence of surface energy ($\Delta\gamma$) with respect to slab thickness for (a) Sn(200) and (b) Bi(012). Energies are referenced to the thickest slab considered ($\Delta\gamma$ = 0). The blue shaded region denotes the estimated thermal noise normalized by the slab area.

Construction of Bi–Sn Heterostructure Slab

Table S4. Relative lattice mismatch for all Bi(012)–Sn(200) in-plane vector matchings. The best integer multiples (m,n) of the Bi and Sn unit vectors and the resulting mismatches are listed.

Matching orientation	Integers (m,n)	Relative mismatch ϵ (%)
$y_{\mathrm{Sn}} \leftrightarrow y_{\mathrm{Bi}}$	(10,7)	0.12
$y_{\mathrm{Sn}} \leftrightarrow x_{\mathrm{Bi}}$	(14,9)	0.82
$x_{\mathrm{Sn}} \leftrightarrow x_{\mathrm{Bi}}$	(5,6)	0.90
$x_{\mathrm{Sn}} \leftrightarrow y_{\mathrm{Bi}}$	(7,9)	1.58

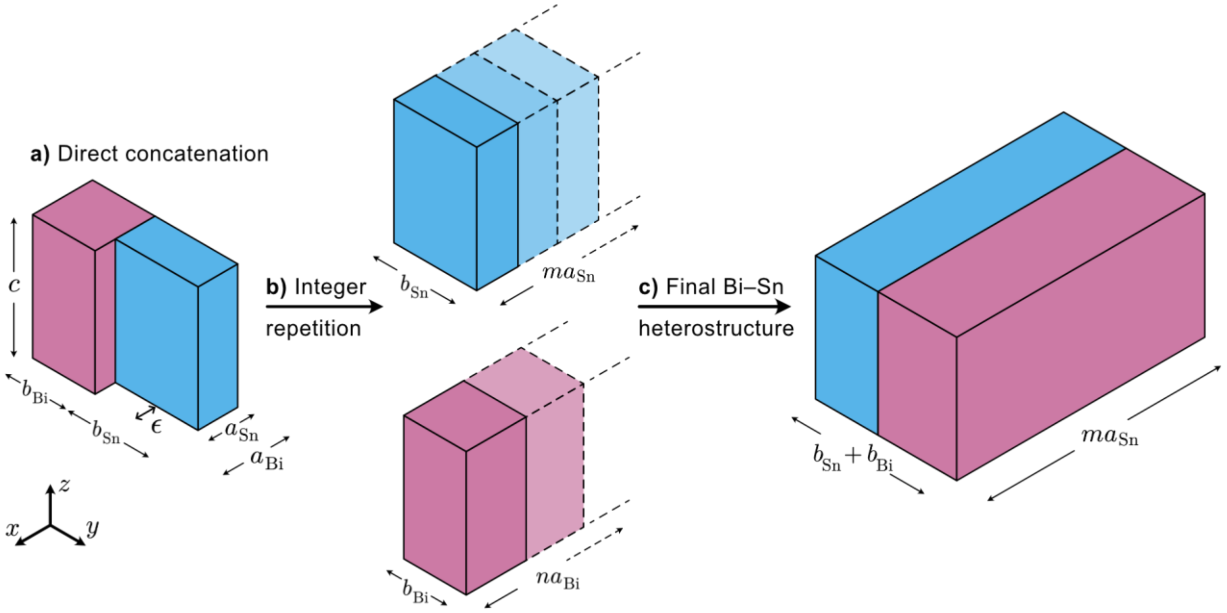

Figure S9. Schematic illustration of the construction of the in-plane Bi–Sn heterostructure slab. (a) Optimized Bi(012) (pink) and Sn(200) (blue) slabs are concatenated along the y-direction, introducing a lattice mismatch, ϵ. Here, a and b denote the in-plane lattice parameters, while c corresponds to the out-of-plane parameter defining the slab vacuum. (b) Integer multiples are applied to minimize the mismatch and form commensurate supercells. The example shown corresponds to commensuration along x and concatenation along y. (c) The commensurate Bi and Sn supercells are then joined laterally to produce the final Bi–Sn heterostructure.

Adsorption Energies of *H on Bi and Sn Surfaces

Table S5. Summary of *H adsorption Gibbs energies per surface atom (in eV) at 0 V_{RHE} ($\Delta g_{nH}(0V_{RHE})$) on Sn and Bi surfaces. θ denotes surface coverage (ML), n the number of adsorbed H atoms, and N_{surf} the number of surface atoms in the supercell. "HER" indicates that hydrogen is evolved during geometry relaxation, marking the coverage limit.

**Metal**	$\boldsymbol{\theta}$	$\boldsymbol{n}$	$\boldsymbol{N_{surf}}$	$\boldsymbol{\Delta g_{nH}(0V_{RHE})}$
Sn	0.25	1	4	0.17
	0.50	1	2	0.35
	0.75	3	4	0.55
	1.00	2	2	0.77
	1.25	3	2	HER
Bi	0.17	1	6	0.15
	0.33	2	6	0.27
	0.50	3	6	0.42

	0.67	4	6	0.55
	0.83	5	6	0.69
	1.00	6	6	0.82
	1.17	7	6	1.06
	1.33	8	6	HER

References

[1] G. Wen, D. U. Lee, B. Ren, F. M. Hassan, G. Jiang, Z. P. Cano, J. Gostick, E. Croiset, Z. Bai, L. Yang, Z. Chen, “Orbital interactions in Bi-Sn bimetallic electrocatalysts for highly selective electrochemical CO_2 reduction toward formate production” Adv. Energy Mater. 2018, 8, 1802427.

[2] J. Tang, R. Daiyan, M. B. Ghasemian, S. A. Idrus-Saidi, A. Zavabeti, T. Daeneke, J. Yang, P. Koshy, S. Cheong, R. D. Tilley, R. B. Kaner, R. Amal, K. Kalantar-Zadeh, “Advantages of eutectic alloys for creating catalysts in the realm of nanotechnology-enabled metallurgy” Nat. Commun. 2019, 10, 4645.

[3] X. An, S. Li, A. Yoshida, T. Yu, Z. Wang, X. Hao, A. Abudula, G. Guan, “Bi-doped SnO nanosheets supported on Cu foam for electrochemical reduction of CO_2 to HCOOH” ACS Appl. Mater. Interfaces 2019, 11, 42114–42122.

[4] Q. Yang, Q. Wu, Y. Liu, S. Luo, X. Wu, X. Zhao, H. Zou, B. Long, W. Chen, Y. Liao, L. Li, P. K. Shen, L. Duan, Z. Quan, “Novel Bi-doped amorphous SnO_x nanoshells for efficient electrochemical CO_2 reduction into formate at low overpotentials” Adv. Mater. 2020, 32, 2002822.

[5] L. Li, A. Ozden, S. Guo, F. P. García de Arquer, C. Wang, M. Zhang, J. Zhang, H. Jiang, W. Wang, H. Dong, D. Sinton, E. H. Sargent, M. Zhong, “Stable, active CO_2 reduction to formate via redox-modulated stabilization of active sites” Nat. Commun. 2021, 12, 5223.

[6] J. Tian, R. Wang, M. Shen, X. Ma, H. Yao, Z. Hua, L. Zhang, “Bi–Sn oxides for highly selective CO_2 electroreduction to formate in a wide potential window” ChemSusChem 2021, 14, 2247–2254.

[7] Y. Zhao, X. Liu, Z. Liu, X. Lin, J. Lan, Y. Zhang, Y.-R. Lu, M. Peng, T.-S. Chan, Y. Tan, “Spontaneously Sn-doped Bi/BiO_x core-shell nanowires toward high-performance CO_2 electroreduction to liquid fuel” Nano Lett. 2021, 21, 6907–6913.

[8] B. Ren, G. Wen, R. Gao, D. Luo, Z. Zhang, W. Qiu, Q. Ma, X. Wang, Y. Cui, L. Ricardez–Sandoval, A. Yu, Z. Chen, “Nano-crumples induced Sn-Bi bimetallic interface pattern with moderate electron bank for highly efficient CO_2 electroreduction” Nat. Commun. 2022, 13, 2486.

[9] Y. Qiao, W. Lai, K. Huang, T. Yu, Q. Wang, L. Gao, Z. Yang, Z. Ma, S. Tulai, M. Liu, C. Lian, H. Huang, “Engineering the local microenvironment over Bi nanosheets for highly selective electrocatalytic conversion of CO_2 to HCOOH in strong acid” ACS Catal. 2022, 12, 2357–2364.

[10] Y. Wang, C. Wang, Y. Wei, F. Wei, L. Kong, J. Feng, J.-Q. Lu, X. Zhou, F. Yang, “Efficient and selective electroreduction of CO_2 to HCOOH over bismuth-based bromide perovskites in acidic electrolytes” Chem. Eur. J. 2022, 28, e202201832.

[11] L.-P. Chi, Z.-Z. Niu, Y.-C. Zhang, X.-L. Zhang, J. Liao, Z.-Z. Wu, P.-C. Yu, M.-H. Fan, K.-B. Tang, M.-R. Gao, “Efficient and stable acidic CO_2 electrolysis to formic acid by a reservoir structure design” Proc. Natl. Acad. Sci. U. S. A. 2023, 120, e2312876120.

[12] L. Li, Z. Liu, X. Yu, M. Zhong, “Achieving high single-pass carbon conversion efficiencies in durable CO_2 electroreduction in strong acids via electrode structure engineering” Angew. Chemie Int. Ed. 2023, 62, e202300226.

[13] H. Shen, H. Jin, H. Li, H. Wang, J. Duan, Y. Jiao, S.-Z. Qiao, “Acidic CO_2-to-HCOOH electrolysis with industrial-level current on phase engineered tin sulfide” Nat. Commun. 2023, 14, 2843.

[14] B. J. Keene, “Review of data for the surface tension of pure metals” Int. Mater. Rev. 1993, 38, 157–192.
